# A *KCNC3* mutation causes a neurodevelopmental, non-progressive SCA13 subtype associated with dominant negative effects and aberrant EGFR trafficking

**DOI:** 10.1371/journal.pone.0173565

**Published:** 2017-05-03

**Authors:** Swati Khare, Jerelyn A. Nick, Yalan Zhang, Kira Galeano, Brittany Butler, Habibeh Khoshbouei, Sruti Rayaprolu, Tyisha Hathorn, Laura P. W. Ranum, Lisa Smithson, Todd E. Golde, Martin Paucar, Richard Morse, Michael Raff, Julie Simon, Magnus Nordenskjöld, Karin Wirdefeldt, Diego E. Rincon-Limas, Jada Lewis, Leonard K. Kaczmarek, Pedro Fernandez-Funez, Harry S. Nick, Michael F. Waters

**Affiliations:** 1 Department of Neurology, University of Florida, Gainesville, FL, United States of America; 2 McKnight Brain Institute, University of Florida, Gainesville, FL, United States of America; 3 Department of Biomedical Engineering, University of Florida, Gainesville, FL, United States of America; 4 Department of Pharmacology, Yale University, New Haven, CT, United States of America; 5 Department of Neuroscience, University of Florida, Gainesville, FL, United States of America; 6 Department of Molecular Genetics and Microbiology, University of Florida, Gainesville, FL, United States of America; 7 Department of Neurology, Karolinska University Hospital, Stockholm, Sweden; 8 Department of Clinical Neuroscience, Karolinska Institute, Stockholm, Sweden; 9 Department of Neurology, Dartmouth-Hitchcock Medical Center, Lebanon, NH, United States of America; 10 Genomics Institute, Multicare Health System, Tacoma, WA, United States of America; 11 Department of Genetics, Karolinska University Hospital, Stockholm, Sweden; 12 Department of Molecular Medicine and Surgery, Karolinska Institute, Center for Molecular Medicine, Stockholm, Sweden; 13 Department of Medical Epidemiology and Biostatistics, Karolinska Institute, Stockholm, Sweden; Universitatsklinikum Wurzburg, GERMANY

## Abstract

The autosomal dominant spinocerebellar ataxias (SCAs) are a diverse group of neurological disorders anchored by the phenotypes of motor incoordination and cerebellar atrophy. Disease heterogeneity is appreciated through varying comorbidities: dysarthria, dysphagia, oculomotor and/or retinal abnormalities, motor neuron pathology, epilepsy, cognitive impairment, autonomic dysfunction, and psychiatric manifestations. Our study focuses on SCA13, which is caused by several allelic variants in the voltage-gated potassium channel KCNC3 (Kv3.3). We detail the clinical phenotype of four SCA13 kindreds that confirm causation of the *KCNC3*^*R423H*^ allele. The heralding features demonstrate congenital onset with non-progressive, neurodevelopmental cerebellar hypoplasia and lifetime improvement in motor and cognitive function that implicate compensatory neural mechanisms. Targeted expression of human KCNC3^R423H^ in *Drosophila* triggers aberrant wing veins, maldeveloped eyes, and fused ommatidia consistent with the neurodevelopmental presentation of patients. Furthermore, human KCNC3^R423H^ expression in mammalian cells results in altered glycosylation and aberrant retention of the channel in anterograde and/or endosomal vesicles. Confirmation of the absence of plasma membrane targeting was based on the loss of current conductance in cells expressing the mutant channel. Mechanistically, genetic studies in *Drosophila*, along with cellular and biophysical studies in mammalian systems, demonstrate the dominant negative effect exerted by the mutant on the wild-type (WT) protein, which explains dominant inheritance. We demonstrate that ocular co-expression of KCNC3^R423H^ with *Drosophila* epidermal growth factor receptor (dEgfr) results in striking rescue of the eye phenotype, whereas KCNC3^R423H^ expression in mammalian cells results in aberrant intracellular retention of human epidermal growth factor receptor (EGFR). Together, these results indicate that the neurodevelopmental consequences of KCNC3^R423H^ may be mediated through indirect effects on EGFR signaling in the developing cerebellum. Our results therefore confirm the *KCNC3*^*R423H*^ allele as causative for SCA13, through a dominant negative effect on KCNC3^WT^ and links with EGFR that account for dominant inheritance, congenital onset, and disease pathology.

## Introduction

Patients with dominant cerebellar ataxias display adult-onset, progressive motor incoordination, and cerebellar atrophy [1–5]. Previously, we reported causation of the autosomal dominant disorder SCA13 by mutations in the voltage-gated potassium channel gene, *KCNC3* (MIM: 176264, Kv3.3) [6–8]. This tetrameric-delayed rectifier channel facilitates rapid firing of action potentials in the cerebellum, hippocampus, and brainstem [9–12]. Two allelic forms of SCA13 [7, 8, 13], p.Arg420His (*KCNC3*^*R420H*^) and p.Phe448Leu (*KCNC3*^*F448L*^) [7], have been described. Their phenotypes are distinct in that *KCNC3*^*R420H*^ results in a slowly progressive, adult-onset ataxia, whereas *KCNC3*^F448L^ presents in childhood with delayed motor milestones. Screening of ataxia DNA repositories identified a third mutation, g.10693G>A; p.Arg423His (*KCNC3*^*R423H*^), displaying early-onset SCA13 [14–16]. We report a detailed phenotypic description of this allelic form in a child who presented at age 7 months and in three additional multigeneration kindreds with multiple affected persons. Features include infantile onset with delayed gross and/or fine motor milestones, tremor, seizures, cognitive impairment, gait and/or appendicular ataxia, and dysarthria. Magnetic resonance imaging (MRI) confirms marked cerebellar hypoplasia as early as 10 months of age. Longitudinal follow-up demonstrates non-progressive cerebellar hypoplasia, with lifetime improvement in motor and cognitive function. Therefore, SCA13^R423H^ may be considered a congenital ataxia causing a fixed deficit, that is, it is partially overcome by normal development, with eventual accomplishment of motor and cognitive milestones. We provide supportive evidence for neurodevelopmental onset through a *Drosophila* model. Biophysical studies and experiments on cellular localization also address channel activity and protein trafficking. Our studies also speak to a cellular basis for dominant inheritance and congenital cerebellar hypoplasia.

## Materials and methods

### Human genotyping and MRI

Patient DNA was isolated from blood (QIAamp Blood kit) or saliva (Oragene) following written informed consent and approval from the Institutional Review Board of the University of Florida, Gainesville, Florida (IRB project 484–2007). Written informed consent for minors was obtained from the next of kin. This study was performed in accordance with the Declaration of Helsinki. All patient-derived sequencing was performed with specific primers (S1 Table) at the DNA Sequencing Core, University of Florida. Midline sagittal T1-weighted MR images of patients were collected at multiple institutions.

### Plasmid constructs

The human *KCNC3*^*WT*^ cDNA was provided by Dr. James L. Rae (Mayo Foundation, Rochester, Minnesota). Individual mutants (*KCNC3*^*R420H*^, *KCNC3*^*R423H*^, or *KCNC3*^*F448L*^) were generated by polymerase chain reaction (PCR) with QuikChange Mutagenesis (Agilent Technologies) (S1 Table). The cDNA encoding human *KCNC3*^*WT*^ and *KCNC3*^*R423H*^ was amplified with primers appropriate for translation in flies, subcloned into the fly expression vector pUAST, then injected into yw embryos (Rainbow Transgenic Flies). All constructs were verified by sequencing. PCR-amplified human *KCNC3*^*WT*^ cDNA and all mutant coding sequences with an enhanced Kozak site were subcloned into modified green fluorescent protein (GFP) and red fluorescent protein (RFP) vectors, pcDNA3-Clover and pcDNA3-mRuby2 (Addgene #40259, #40260, provided by Michael Lin) [17], or Cerulean3-N1 [18] (Addgene #54742, provided by Michael Davidson, S1 Table). Human perbB1-Citrine (EGFR^Citrine^) was purchased from Addgene (#40266), courtesy Martin Offterdinger. GFP-JMY-Full length (GFP-junction-mediating and regulatory protein [JMY^GFP^]) and GFP-PI4K2A wild-type (PI4K2A^GFP^) were provided by Theresia E.B. Stradal (Helmholtz Centre for Infection Research, Braunschweig, Germany) and Tamas Balla (National Institute of Child Health and Human Development, National Institutes of Health), respectively. N-Cadherin-EGFP (Addgene plasmid # 18870) was provided by Valeri Vasioukhin.

### *Drosophila* genetics and imaging

Fly stocks were obtained from the Bloomington *Drosophila* Stock Center (flystocks.bio.indiana.edu, S2 Table). All crosses were at 25°C unless indicated otherwise. Adult wings (n = 6) and frozen adult eyes (n = 7) were processed and imaged, as previously described [19].

### Cell culture and transfection

Flp-In-CHO (Chinese hamster ovary, Thermo Fisher Scientific) and human glioblastoma U87 cells (ATCC, HTB-14) were cultured in Ham’s F-12 or DMEM (Dulbecco’s modified Eagle’s medium) (Corning), respectively, with 10% fetal bovine serum (FBS), 25mM glucose, 4mM glutamine, and pen/strep (penicillin-streptomycin) at 37°C, 5% CO_2_. Transient transfections were carried out using Lipofectamine LTX with Plus reagent (Life Technologies). Total DNA was kept constant by adding control pcDNA3.1 plasmid.

### Immunoblot analysis

Protein from individual 2-day-old flies or CHO or U87 cells was analyzed, as previously described, [19] with the modification of resolution with 3–8% Tris-acetate gels (Life Technologies). Co-immunoprecipitation experiments were performed with Dynabeads Protein G (Thermo Fisher Scientific) using α-EGFR (Abcam) for binding and α-EGFR (EMD Millipore) and α-KCNC3 (Alomone Labs) for detection. Full-length blots are presented in S1 Fig.

### Electrophysiology

CHO cells were grown in Iscove’s modified Dulbecco’s medium (Invitrogen), with 10% FBS, 100 units/ml pen/strep, 5% HT supplement (Thermo Fisher Scientific) in 5% CO_2_ at 37°C. Seeded cells were transfected 24h later using Lipofectamine reagent (Invitrogen) with the mRuby2- or Clover-tagged KCNC3^WT^ or KCNC3^R423H^ constructs. On the recording day, patch electrodes were pulled from 1.5mm outside diameter borosilicate glass capillaries (World Precision Instruments). The resistance of a typical electrode was 2-3M for whole-cell recording when filled with intracellular solution (in mM) of 97.5 potassium gluconate, 32.5 KCl, 10 HEPES, 5 EGTA, and pH7.2, with KOH. The bath solution consisted of (in mM) 140 NaCl, 5.4 KCl, 1.3 CaCl_2_, 25 HEPES, 33 glucose, and pH 7.4, with NaOH. Series resistance was 2-4M and was compensated by 80–85%. The data were acquired at 10 kHz and filtered at 5 kHz, using pClamp8 software (Molecular Devices).

### Immunofluorescence

Eye-imaginal discs of third instar fly larvae were immunostained using a standard protocol [20] with bovine serum albumin (BSA) for blocking. Rat α-elav (7E8A10, 1:200), developed by Gerald M. Rubin, and mouse α-chaoptin (24B10, 1:200), developed by S. Benzer and N. Colley, were obtained from the Developmental Studies Hybridoma Bank (University of Iowa, Iowa City, Iowa). CHO cells were transiently transfected with pBKCMV-*KCNC3*^*WT*^, pBKCMV-*KCNC3*^*R420H*^, and pBKCMV-*KCNC3*^*R423H*^ plasmids, grown on poly-D-lysine–coated coverslips, fixed (4% formaldehyde, 20 min), permeabilized (0.1% Triton X-100, 10 min) and blocked with 10% BSA. The samples were stained overnight with α-KCNC3 (1:500, Alomone Labs) at 4°C, washed with PBS, and subjected to secondary-antibody (Alexa Fluor 594, anti-rabbit, Molecular Probes) for 1h with samples without the primary processed simultaneously. CHO cells transiently transfected with Clover-, mCerulean3-, or mRuby2-tagged *KCNC3*^*WT*^, *KCNC3*^*F448L*^, *KCNC3*^*R420H*^, and *KCNC3*^*R423H*^ vectors were imaged 48h post-transfection in Live Cell Imaging Solution (Molecular Probes) using a Nikon A1 confocal or inverted Olympus IX-HLSH100 microscope. Images were processed using NIS-Elements (Nikon) and Adobe Photoshop CS6.

### Statistical analysis

Statistical analysis with SEM values are presented using an unpaired, two-tailed t-test for significance (***, p<0.001). All analyses were performed using Microsoft Excel and GraphPad Prism.

## Results

### Causative inheritance, phenotype and MR imaging associated with the *KCNC3*^*R423H*^ allele

**Fig 1:**
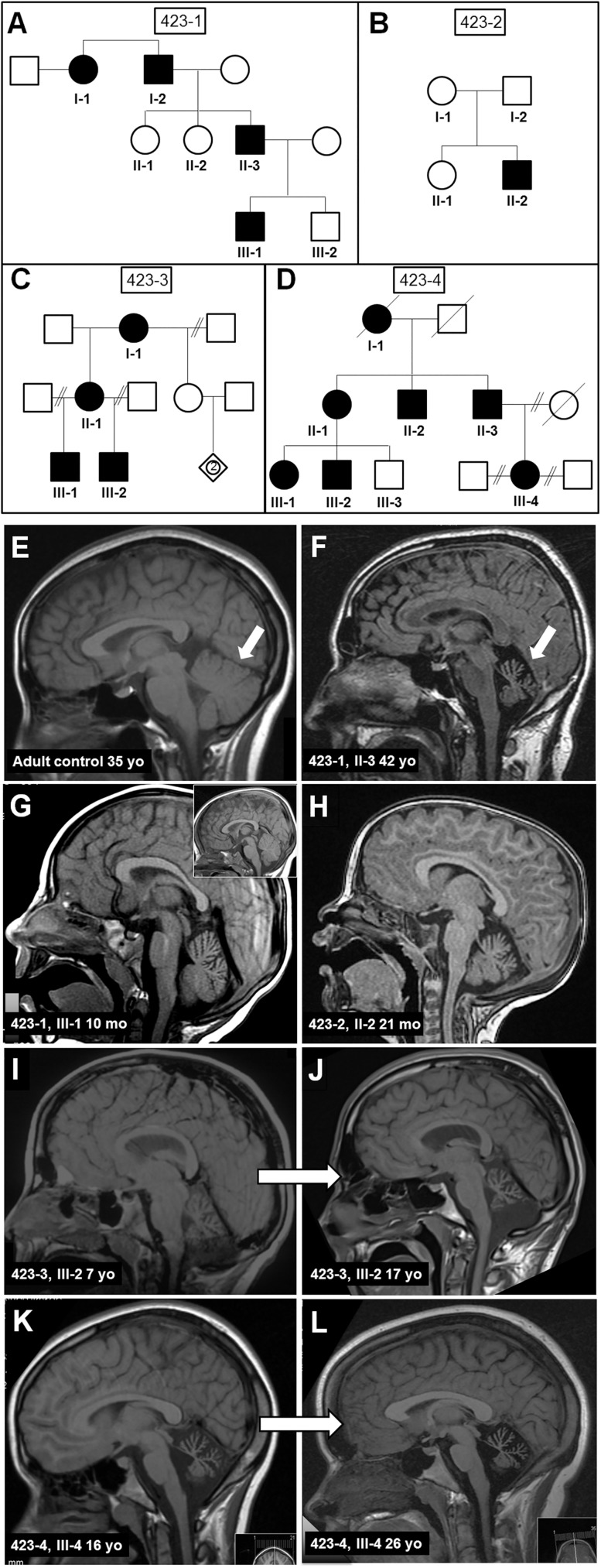
Pedigrees of the probands’ families and MRI. Four pedigrees (A) 423–1, (B) 423–2, (C) 423–3, and (D) 423–4, illustrating the inheritance pattern of KCNC3^R423H^. De novo inheritance in patient II-2 is illustrated in 423–2. Midline T1-weighted sagittal magnetic resonance images (MRIs) of (E) a 35-year-old control; (F) patient 423–1, II-3 at age 42 years; (G) patient 423–1, III-1 at age 10 months (inset shows age-matched control); (H) de novo patient 423–2, II-2 at age 21 months. Midline T1-weighted sagittal MRIs of (I,J) patient 423–3, III-2 at age 7 and 17 years, respectively; and (K,L) patient 423–4, III-4 at age 16 and 26 years, respectively, demonstrating the lack of progressive cerebellar hypoplasia and/or atrophy.

Four multigenerational pedigrees segregating *KCNC3*^*R423H*^ provided definitive genetic proof of causation through complete fidelity in allele segregation of sequenced, affected, and unaffected family members (Fig 1A–1D). Case 423–2 illustrates de novo inheritance in patient II-2. Midline T1-weighted sagittal MR images demonstrate pronounced cerebellar hypoplasia. A 35-year-old control (Fig 1E) is compared to a 42-year-old affected family member (423–1, II-3, Fig 1F). T1-weighted MR images of a 10-month-old (423–1, III-1, Fig 1G; inset; age-matched control) and a 21-month-old (423–2, II-2, Fig 1H) demonstrate severe cerebellar hypoplasia, consistent with neurodevelopmental onset. Patient 423–2, II-2 presented with a seizure at 7 months and was diagnosed by genotyping and head CT. Review of available patient histories confirms infantile onset.

The MR images for individuals 423–3, III-2 at 7 and 17 years (Fig 1I and 1J) and 423–4, III-4 at 16 and 26 years (Fig 1K and 1L) similarly illustrate severe cerebellar hypoplasia. Strikingly, these two individuals display minimal age-dependent atrophy in 10-year serial scans (compare Fig 1I to 1J; 1K to 1L). This contrasts to the *KCNC3*^*R420H*^ allele where patients demonstrate marked progression of cerebellar atrophy over time with concomitant symptom progression [8]. Furthermore, individual III-2 (423–3, Fig 1C, 1I and 1J) was wheelchair-bound during early adolescence, transitioning to a walker, then to a cane, and ambulating independently by late teens, although not completely normally. Patient III-4 (423–4, Fig 1D, 1K and 1L) was initially diagnosed with cerebral palsy and moderate cognitive impairment, but progressed to unassisted ambulation and/or running and normal-range cognition by age 26 years. A motor evaluation in 2013 yielded a SARA (Scale for the Assessment and Rating of Ataxia) score [21] of 13, which remained unchanged at12-month follow-up. Both patients therefore display non-progressive cerebellar hypoplasia, along with displaying many other activity-dependent improvements in motor and cognitive milestones. Phenotypic data and ataxic parameters (S3 and S4 Tables), demonstrate uniformity in clinical features within and across families. These kindreds establish several novel aspects not typical of other SCAs [4, 22–24]: a neurodevelopmental pattern with infantile onset, non-progression of cerebellar atrophy, and clinical symptoms accompanied by cognitive and motor improvement suggestive of compensatory neural mechanisms despite severe cerebellar hypoplasia.

### Expression of human KCNC3^R423H^ in *Drosophila melanogaster* wing and eye

**Fig 2:**
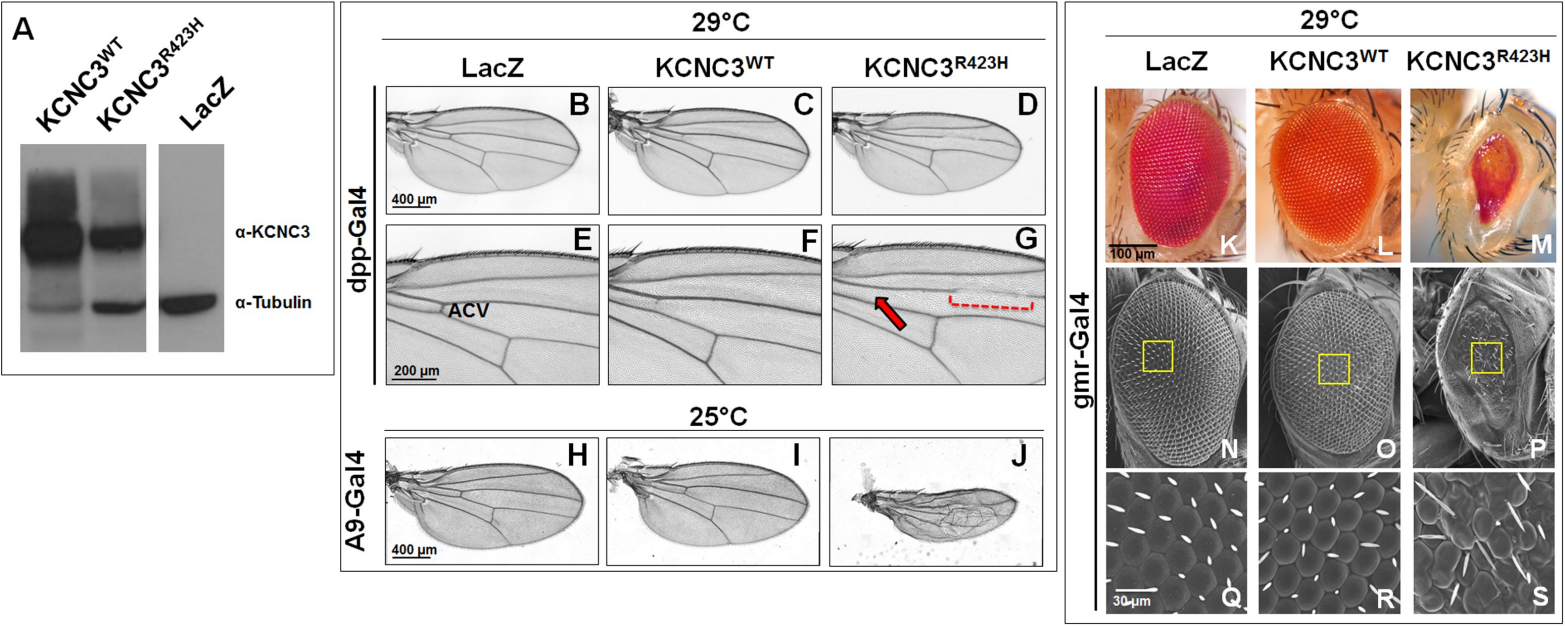
Expression of human *KCNC3*^WT^ and *KCNC3*^R423H^ in the *Drosophila* wing and eye. (A) Cropped immunoblot using antibodies directed against mammalian *KCNC3* and *Drosophila* tubulin illustrates da-Gal4 expression of the indicated transgenes (human *KCNC3*^*WT*^, *KCNC3*^*R423H*^, or β-galactosidase (*LacZ*)) under control of the ubiquitous da-Gal4 driver (full-length blot shown in S1 Fig). (B-D) Wing images from adult flies maintained at 29°C, where the dpp-Gal4 drives expression of LacZ, KCNC3^WT^, or KCNC3^R423H^ in the anterioposterior border of the wing. (E-G) Magnified images showing loss of the anterior crossvein (ACV [E]; red arrow [G]) and disruption of the longitudinal vein L3 as a consequence of KCNC3^R423H^ expression (dotted brackets [G]). (H-J) Wing images from adult flies where A9-Gal4 drives expression in the wing compartment. (K-M) Eye images from adult flies where gmr-Gal4 drives expression of LacZ, KCNC3^WT^, or KCNC3^R423H^, demonstrating small, maldeveloped eyes in the mutant. (N-P) Scanning electron microscopy images of whole eyes from *LacZ*, *KCNC3*^*WT*^, or *KCNC3*^*R423H*^ flies. Yellow square highlights region of eye shown at high resolution (Q-S).

Developmental consequences of R423H expression were examined in strains of *Drosophila melanogaster* expressing human KCNC3^WT^ and KCNC3^R423H^ controlled by the yeast UAS/Gal4 expression system [25]. All driver and responder fly strains are summarized in S2 Table. Expression of KCNC3^WT^ and KCNC3^R423H^ in transgenic flies was verified by immunoblot (Fig 2A) from individual 2-day-old flies controlled by the ubiquitous daughterless (da)-Gal4 driver [26]. We expressed LacZ, KCNC3^WT^, or KCNC3^R423H^ in the developing wing (Fig 2B–2G), along the anteroposterior boundaries extending to the anterior compartment using the decapentaplegic (dpp)-Gal4 driver. Gal4/KCNC3^R423H^ expression caused complete loss of the anterior cross vein (ACV) and partial loss of vein L3 (Fig 2G). The flies were kept at 29°C to achieve maximal Gal4-transgene expression with minimal effects on viability and fertility [27]. KCNC3^R423H^ expression in the developing wing pouch, using the A9-Gal4 driver [28], resulted in severely altered wing morphology and vein patterning (Fig 2H–2J).

To test the mutant allele in a developing neuronal lineage, we utilized the eye-specific driver gmr-Gal4 [29] for expression throughout eye development. KCNC3^R423H^ expression displayed marked eye dysmorphology and a profound reduction in size (Fig 2M), disrupted ommatidial organization, fused ommatidia, and malformed eye bristles (Fig 2P and 2S) compared to those of a normal eye phenotype in *KCNC3*^*WT*^ and a control *LacZ* (Fig 2K, 2L, 2N, 2O, 2Q and 2R). Sagittal sections of the *KCNC3*^*WT*^ eyes illustrate regular arrangement of each lens at the apex of the elongated ommatidium, in contrast with the disrupted pattern observed in the *KCNC3*^*R423H*^ eyes (S2A and S2B Fig). In addition to the adult eye, we stained the eye imaginal discs from wandering third instar larvae for the pan-neuronal marker elav [30]. Control *KCNC3*^*WT*^ larval imaginal discs revealed expected organized ommatidia (S2C Fig), whereas *KCNC3*^*R423H*^ larvae showed disorganized and fused ommatidia with maldeveloped smaller ommatidial clusters (S2D Fig). Detection of chaoptin, a photoreceptor cell- and axon-specific membrane protein required for cell morphogenesis [31], illustrates normal organization of the axon bundle from the ommatidia to the brain in the *KCNC3*^*WT*^ larvae (S2E Fig), whereas *KCNC3*^*R423H*^ larvae displayed disorganized and diminished axonal projections (S2F Fig).

### KCNC3^R423H^ intracellular location and biophysics

**Fig 3:**
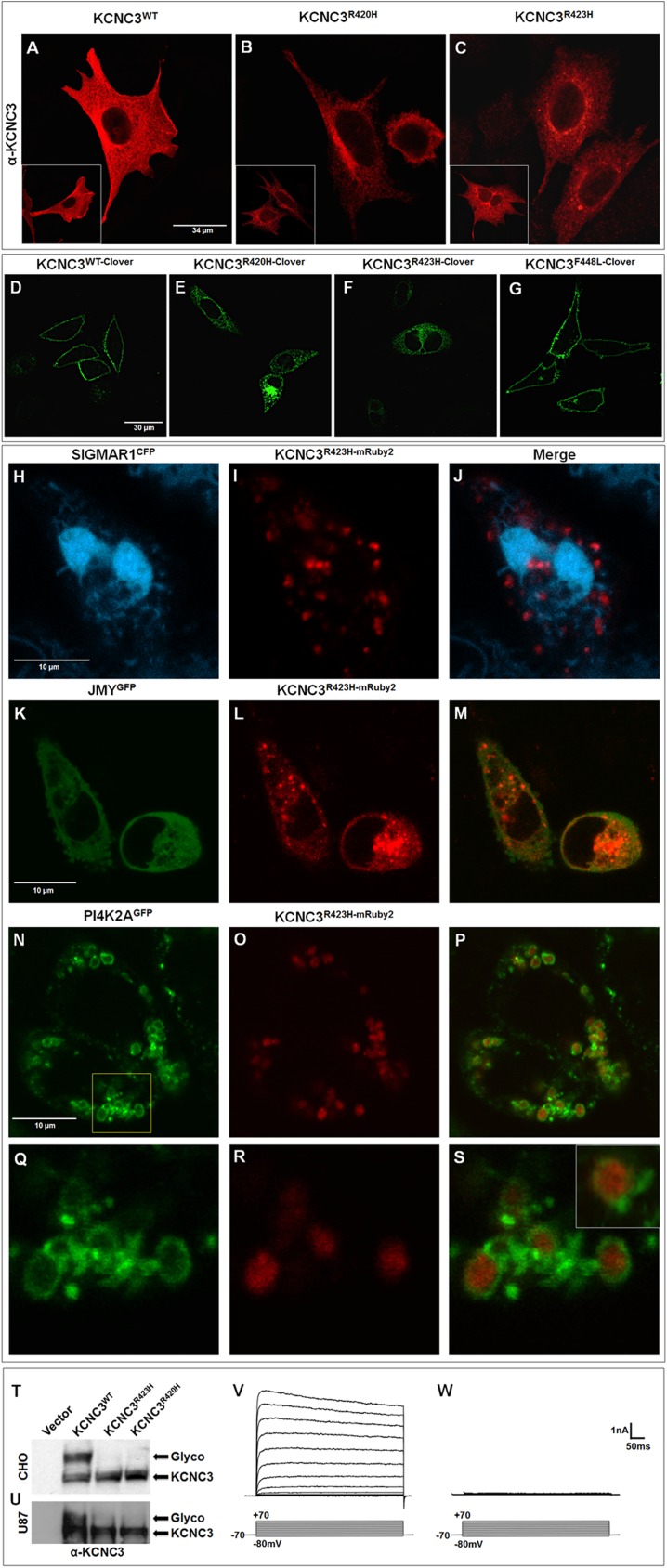
KCNC3^R423H^ displays aberrant intracellular trafficking, glycosylation, and failure to express current in cell culture. (A-C) Immunofluorescence of CHO cells expressing human KCNC3^WT^, KCNC3^R420H^, or KCNC3^R423H^ using a KCNC3 antibody. Insets illustrate other representative cells from each transfection. (D-G) Confocal fluorescence images of CHO cells transiently expressing Clover-tagged human KCNC3^WT^, KCNC3^R420H^, KCNC3^R423H^, or KCNC3^F448L^. Fluorescence images of CHO cells transiently expressing (H-J) an ER marker, SIGMAR1^CFP^ with KCNC3^R423H-mRuby2^; (K-M) an anterograde vesicle marker, JMY^GFP^ with KCNC3^R423H-mRuby2^; and (N-P) a Golgi–endosome vesicle marker, PI4K2A^GFP^ with KCNC3^R423H-mRuby2^. (N) Boxed area is magnified in (Q-S), with inset in S clearly demonstrating intravesicular retention of KCNC3^R423H-mRuby2^. Cropped immunoblot of human KCNC3^WT^, KCNC3^R423H^, or KCNC3^R420H^ expressed in (T) CHO cells or (U) human U87 glioblastoma cells illustrating aberrant glycosylation for both causative mutant alleles (full-length blot shown in S1 Fig). Representative currents evoked by commands to potentials between −80 mV and +70 mV, recorded in CHO cells expressing (V) KCNC3^WT-Clover^ or (W) KCNC3^R423H-Clover^.

We recently reported that the *KCNC3*^*R420H*^ allele [7, 8, 32] displayed altered post-translational modifications with aberrant retention in the Golgi [19]. CHO cells, with no detectable endogenous KCNC3, transiently transfected with human *KCNC3*^*WT*^ (Fig 3A), show normal plasma membrane localization by immunofluorescence. In contrast, cells expressing KCNC3^R423H^ (Fig 3C; compare to KCNC3^R420H^ in Fig 3B), demonstrate aberrant trafficking with strong perinuclear staining analogous to the *KCNC3*^*R420H*^ allele [19]. As illustrated with other representative cells (Fig 3B and 3C insets) aberrant trafficking is consistently observed with both mutants. To corroborate the aberrant trafficking, we then generated C-terminal Clover-tagged vectors [17] harboring. Confocal microscopy illustrates normal plasma membrane localization (Fig 3D) for KCNC3^WT^, in contrast to absent plasma membrane localization and clear perinuclear retention for each mutant (Fig 3E and 3F). This aberrant trafficking is found in all cells from multiple independent transfection experiments. For further comparison, we also expressed the Clover-tagged SCA13 mutation from a French pedigree, *KCNC3*^*F448L*^, which displays normal plasma membrane trafficking (Fig 3G), although associated with altered biophysical properties [7].

To demonstrate that protein overexpression was not responsible for aberrant localization, we show that over about an 8-fold range of transfected plasmid concentrations, with vectors harboring human *KCNC3*^*WT*^ and *KCNC3*^*R423H*^ C-terminally tagged with mCerulean3 [18], KCNC3^R423H-mCerulean3^ remains intracellularly localized, with no detectable plasma membrane trafficking, in contrast to normal localization with equivalent concentrations of the *KCNC3*^*WT*^ allele (S3 Fig). This finding clearly demonstrates that channel mis-trafficking is not attributable to protein expression levels.

To determine the intracellular localization of the mutant channel, we used fluorescently tagged markers of endoplasmic reticulum (ER), Golgi, and intracellular vesicles. Sigma receptor 1 (SIGMAR1) is a highly conserved, transmembrane chaperone protein located in the ER membrane [33]. On co-expression of the cyan fluorescent protein (CFP) tagged SIGMAR1 (SIGMAR1^CFP^) with KCNC3^R423H-mRuby2^ (Fig 3H–3J), we observed no co-localization, which rules out aberrant retention in the ER. JMY is linked to cytosolic actin assembly and acts as a DNA damage-induced transcriptional co-activator of p53 [34, 35]. Recently, Schlüter et al. [36] also demonstrated JMY’s role in vesicular trafficking at the *trans*-Golgi network through actin-dependent elongation and/or tubulation of anterograde vesicles. Co-expression of JMY^GFP^ with KCNC3^R423H-mRuby2^ resulted in co-localization (Fig 3K–3M) in vesicular structures, implying the retention of KCNC3^R423H^ in trafficking vesicles. PI4K2A, a membrane-bound phosphatidylinositol-4 kinase, localizes to the *trans-*Golgi network (TGN) and early endosomes [37, 38] and is responsible for phosphorylation of phosphatidylinositol (PI) to phosphatidylinositol 4-phosphate (PI4P), a critical lipid in endocytosis, Golgi function, protein sorting, and membrane trafficking [39]. Co-expression of KCNC3^R423H-mRuby2^ with PI4K2A^GFP^ clearly demonstrates that the mis-trafficked mutant channel accumulates in the intravesicular space of PI4K2A^GFP^–positive vesicles (Fig 3N–3S). These results suggest that KCNC3^R423H^ is retained in the interior of anterograde or endosomal vesicles rather than being incorporated into the vesicular membrane.

As an integral membrane protein, N-glycans are added to the nascent KCNC3 protein in the ER with trimming of the oligosaccharide precursor, a critical quality-control measure for proper glycoprotein folding and vesicular trafficking through the Golgi en route to the plasma membrane. The expression of human KCNC3^R423H^ in CHO (Fig 3T) or U87 cells (Fig 3U) demonstrates altered glycosylation patterns consistent with our previous studies on KCNC3^R420H^ [19].

As a voltage-dependent potassium channel, KCNC3/Kv3.3 displays high activation thresholds with fast activation and deactivation kinetics, conveying the property of sustained trains of high-frequency action potentials in neurons expressing them [40]. To assess the functional consequences of the KCNC3^R423H^ mutation, we performed electrophysiology in CHO cells transiently expressing the human KCNC3^WT^ or KCNC3^R423H^ channels. For KCNC3^WT^, Fig 3V illustrates the slow inactivating outward current evoked during application of depolarizing voltage steps (increments of 10 mV from −80 mV to +70 mV). In contrast, cells expressing the KCNC3^R423H^ channels demonstrate the complete absence of current conductance by the mutant channel over the same voltage range (Fig 3W), consistent with aberrant glycosylation and intracellular retention.

### KCNC3^R423H^ has a dominant effect on KCNC3^WT^

**Fig 4:**
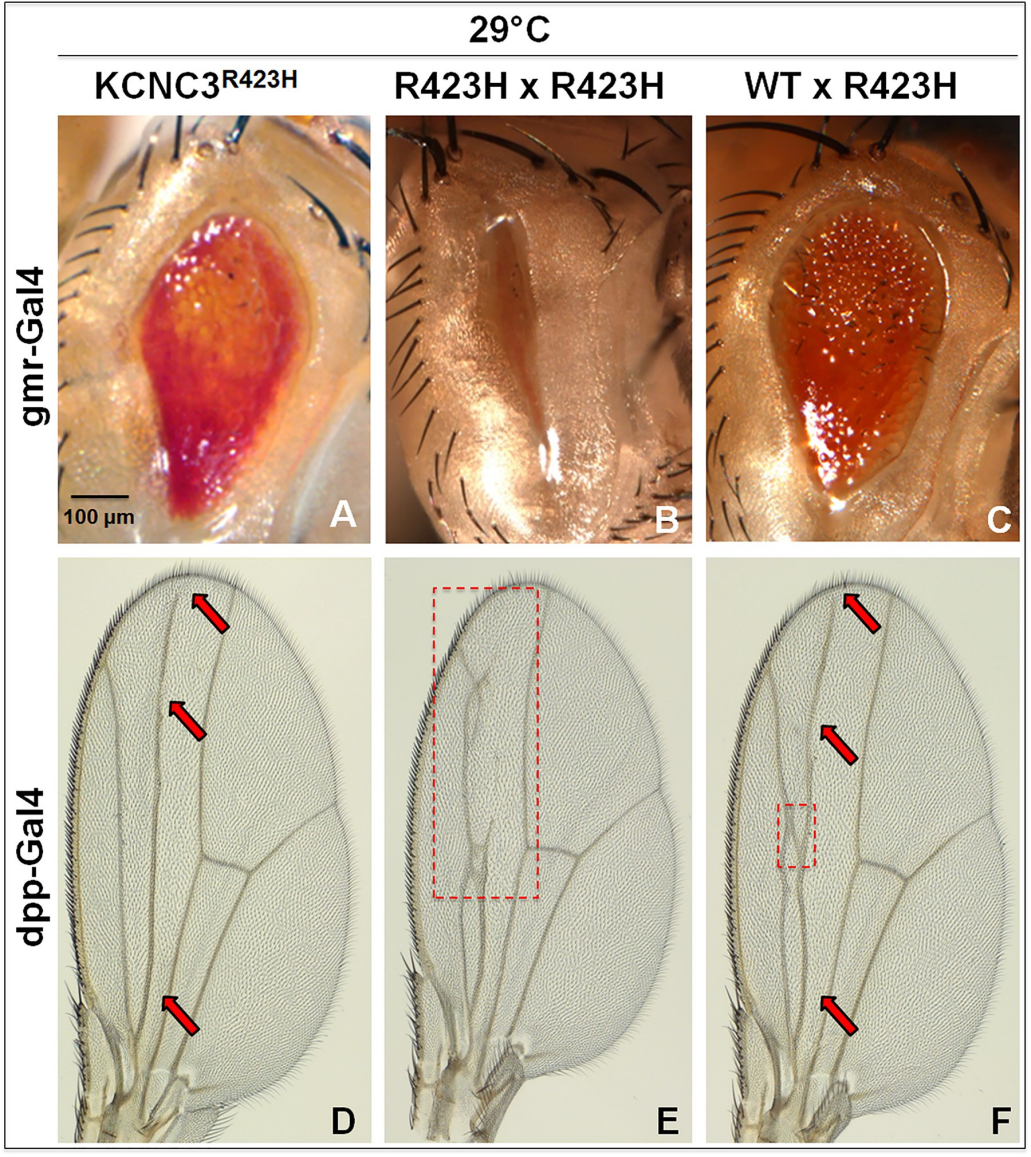
KCNC3^R423H^ causes dominant effects on KCNC3^WT^ in *Drosophila*. Eye images from gmr-Gal4 flies expressing (A) KCNC3^R423H^; (B) two copies of *KCNC3*^*R423H*^; and (C) *KCNC3*^*WT*^ with *KCNC3*^*R423H*^. Wing images from dpp-Gal4 flies expressing (D) KCNC3^R423H^; (E) two copies of *KCNC3*^*R423H*^; and (F) *KCNC3*^*WT*^ with *KCNC3*^*R423H*^. Red arrows and boxes (dashed lines) indicate areas of aberrations.

The consequences of an increased dosage of the *KCNC3*^*R423H*^ allele were determined by creating flies harboring 2× copies of *KCNC3*^*R423H*^, resulting in a more severe eye phenotype entirely lacking ommatidia (Fig 4B compared to Fig 4A). Moreover, simultaneous expression of exogenous KCNC3^WT^ with KCNC3^R423H^ did not overcome the eye phenotype (Fig 4C compared to Fig 4A), which implicates the dominant nature of the R423H allele. Similarly, *KCNC3*^*R423H*^ self-cross, driven by dpp-Gal4 in the wing, led to disruption of veins L2, L3, and ACV in the wing (Fig 4E compared to Fig 4D), whereas co-expression of KCNC3^WT^ with KCNC3^R423H^ resulted in a phenotype similar to KCNC3^R423H^ alone (Fig 4F compared to Fig 4D). Studies in the eye and wing thus support the strong dominant negative effects exerted by *KCNC3*^*R423H*^.

**Fig 5:**
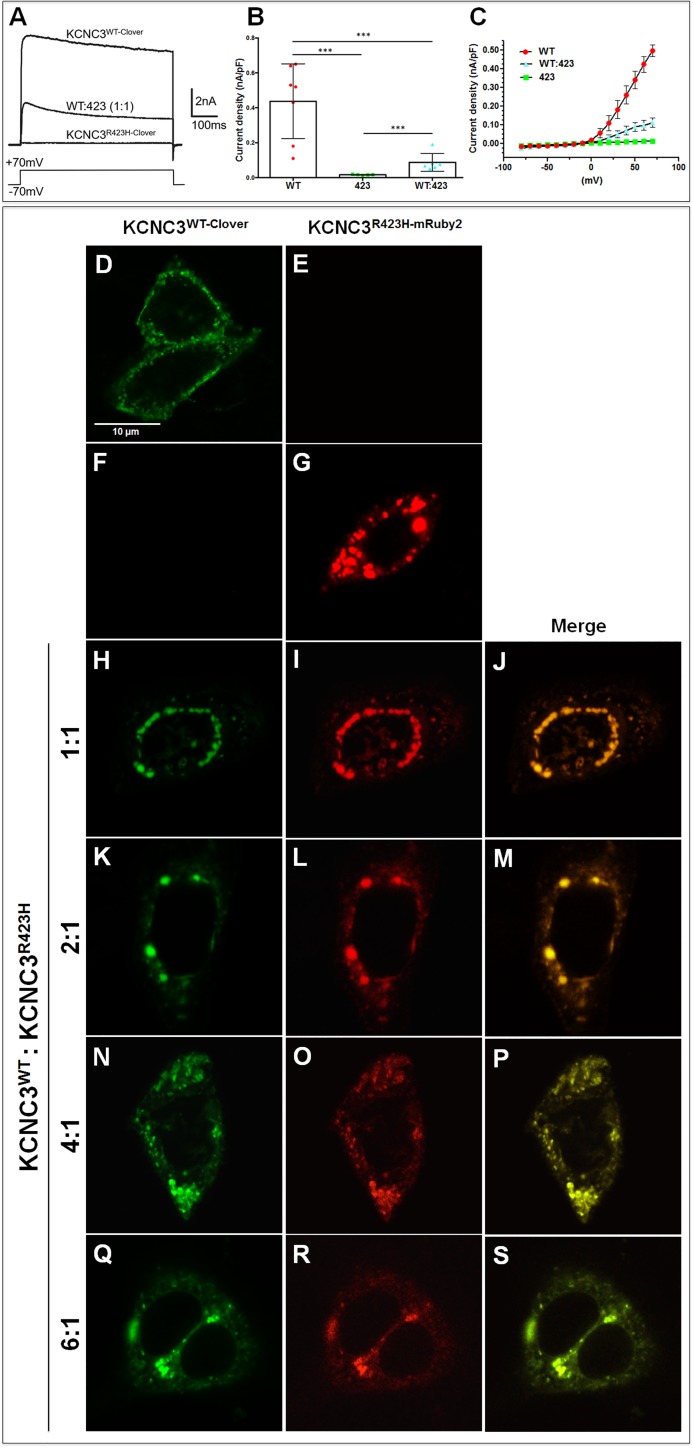
KCNC3^R423H^ causes dominant electrophysiological and trafficking effects on KCNC3^WT^. (A) Representative currents evoked by a step from −70 mV to +70 mV in CHO cells expressing KCNC3^WT-Clover^ or KCNC3^R423H-Clover^, and in those transfected with both constructs KCNC3^WT-Clover^:KCNC3^R423H-mRuby2^ in a 1:1 ratio. (B) Mean current densities recorded in CHO cells expressing either wild-type KCNC3^WT-Clover^ (n = 7) or KCNC3^R423H-Clover^ (n = 5) and in those expressing both KCNC3^WT-Clover^: KCNC3^R423H-mRuby2^ (1:1) constructs (n = 6). Current density was calculated by dividing the peak current evoked by a step from −70 to +70 mV by cell capacitance. Values are shown as mean±SEM, and significance was tested using a one-way ANOVA. (C) Current-voltage relations for cells in the three conditions shown in (A) and (B). Confocal fluorescence microscopy of cells expressing KCNC3^WT-Clover^ (D) or KCNC3^R423H-mRuby2^ (G) individually, with no channel bleed-through (E,F). (H-S) Confocal fluorescence microscopy of cells co-expressing KCNC3^WT-Clover^ and KCNC3^R423H-mRuby2^ at ratios of 1:1 to 6:1 (*KCNC3*^*WT*^:*KCNC3*^*R423H*^) showing co-localization and intracellular retention of both proteins, even at the highest concentration of *KCNC3*^*WT*^. The total amount of DNA used in the co-transfection experiments was kept constant across ratios by adding control plasmid pcDNA 3.1.

KCNC3 functions as a tetrameric voltage-gated potassium channel [41, 42]. This disease presents as an autosomal dominant phenotype, [7, 32] presumably resulting from the formation of a heterotetramer composed of WT and mutant monomers. We have determined the probabilities of heterotetramer interactions based on WT:423 ratios of 1:1 to 6:1 (S4A and S4B Fig). Biophysical studies of CHO cells co-expressing KCNC3^WT^: KCNC3^R423H^ at a ratio of 1:1 demonstrate a significant reduction in current amplitude and mean current densities compared to those of cells expressing KCNC3^WT^ alone (Fig 5A, 5B and 5C), thereby showing a dominant electrophysiological effect. Studies using other model systems have implied that the dominant phenotypic effects manifested by *KCNC3*^*R423H*^ and *KCNC3*^*R420H*^ mutations are based on alterations in channel electrophysiology [43]. These results are not supported by our data [19] (Fig 3A–3S), which demonstrate that neither mutation is appreciably trafficked to the plasma membrane. To address the mechanism underlying the dominant phenotypic effects of *KCNC3*^*R423H*^, we also used differentially C-terminally labeled mutant and WT channels to explore intracellular trafficking and tetrameric protein association. KCNC3^WT-Clover^ was co-expressed with KCNC3^R423H-mRuby2^ in cells at ratios from 1:1 to 6:1 and visualized by confocal microscopy (Fig 5D–5S). As expected, KCNC3^WT-Clover^ alone traffics normally to the plasma membrane (Fig 5D), whereas KCNC3^R423H-mRuby2^ alone displays complete retention in intracellular vesicles (Fig 5G). Co-expression with increasing ratios of KCNC3^WT^: KCNC3^R423H^ demonstrates complete co-localization (Fig 5H–5S) suggestive of tetrameric co-assembly, along with complete retention of the KCNC3^WT^ protein in the same intracellular vesicles even at a ratio of 6:1 (Fig 5Q–5S).

### dEgfr rescues the *Drosophila KCNC3*^*R423H*^ eye phenotype

**Fig 6:**
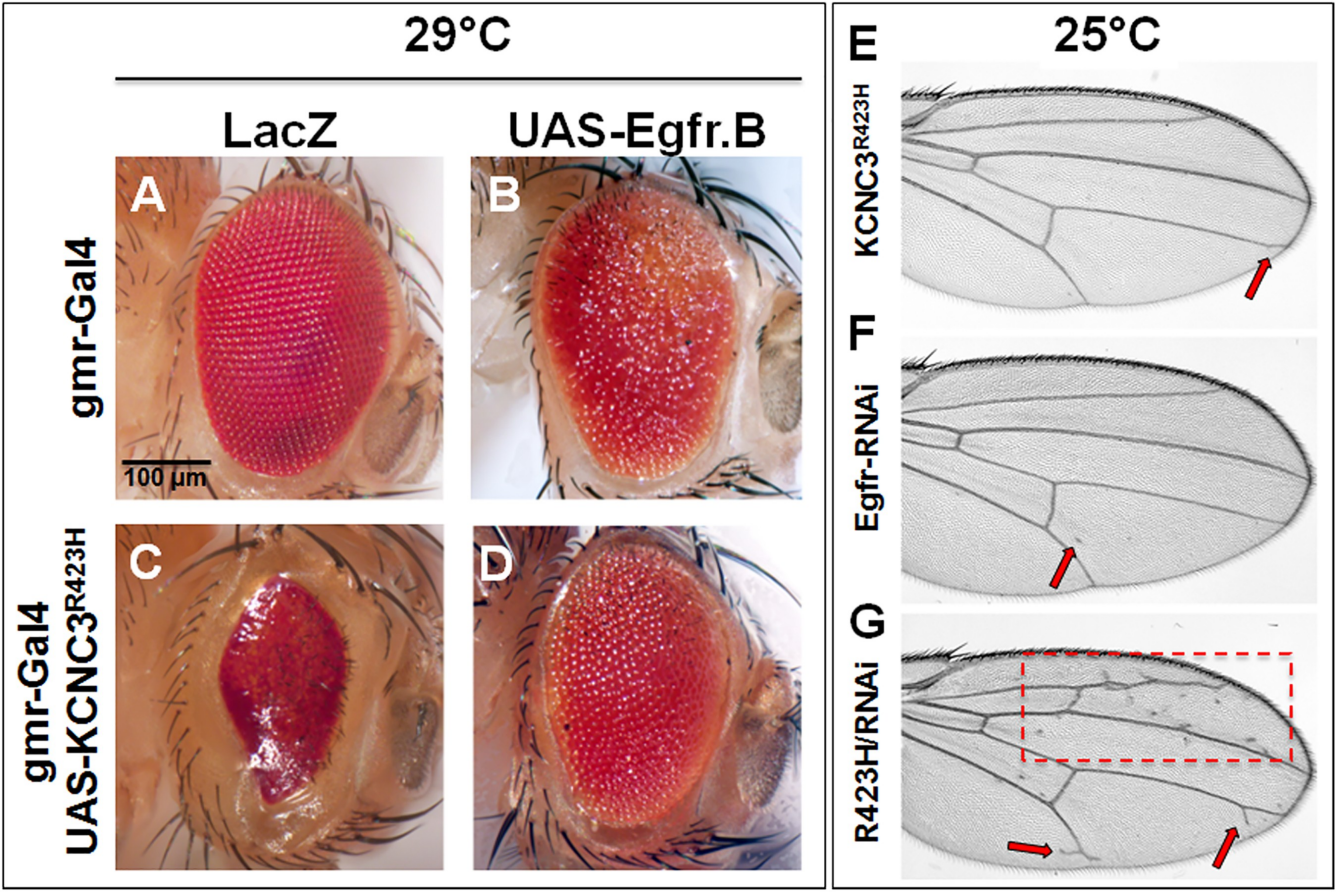
Effects of Egfr on the *KCNC3*^*R423H*^ *Drosophila* eye and wing phenotypes. (A-C) gmr-Gal4–driven expression of controls LacZ, Egfr, and KCNC3^R423H^ individually. (D) Co-expression of Egfr and KCNC3^R423H^ shows rescue of the mutant phenotype. (E-G) dpp-Gal4–driven expression of KCNC3^R423H^ and Egfr-RNAi, individually and co-expressed. Red arrows and boxes (dashed lines) indicate areas of aberrations.

To identify the *Drosophila* pathway(s) affected by *KCNC3*^*R423H*^, we evaluated modifiers of the mutant phenotypes upon co-expression with a series of eye and wing specific determinants, including *dEgfr*, *Ras*, *Rolled* (mitogen-activated protein kinase [MAPK]) and *Notch* [44–46] (S2 Table). Effects of Egfr overexpression (UAS-*Egfr*.B) [47] were evaluated on a gmr-Gal4 (Fig 6A and 6B) or gmr-Gal4, UAS-*KCNC3*^*R423H*^ background (Fig 6C and 6D). Elevated expression of dEgfr with the UAS-*Egfr*.B allele resulted in a striking rescue of the *KCNC3*^*R423H*^ eye phenotype (Fig 6D), which supports a link between the mutant voltage-gated potassium channel and Egfr or its downstream signaling pathway. To further demonstrate interactions between KCNC3^R423H^ and Egfr in eye development, we expressed two *dEgfr* RNAi strains in the context of *KCNC3*^*R423H*^ at 25°C (S5A–S5F Fig). Due to lower Gal4 expression at 25°C, the *KCNC3*^*R423H*^ allele displayed no overt eye pathology (S5D Fig), with the *dEgfr* RNAi strains displaying only minor effects on ommatidia organization (S5B and S5C Fig). However, co-expression of KCNC3^R423H^ and *dEgfr* RNAi transgenes resulted in a reduction in eye size (S5E and S5F Fig) reminiscent of the *KCNC3*^R423H^ phenotype at 29°C (Fig 2M).

Egfr signaling in *Drosophila* involves a linear pathway with equivalent mammalian orthologs as summarized in S6 Fig. We thus evaluated overexpression of downstream mediators Ras (S7B and S7F Fig) and Rolled (MAPK, S7C and S7G Fig), which displayed little effect on the *KCNC3*^*R423H*^ eye phenotype. These data imply that the mutant channel may be impairing Egfr signaling at the level of the receptor, consistent with *KCNC3*^R423H^ dominant effects (Figs 4 and 5). During the third instar larval stage, cells arrest in the eye imaginal disc and constrict at the disc’s posterior edge to form the morphogenetic furrow (MF) [48, 49]. The MF then travels anteriorly, where Notch signaling establishes specification of the initial R8 photoreceptor neurons as the founding cells of the developing ommatidia. Signaling from the Egfr pathway leads to the recruitment of the remaining photoreceptor cells in a pairwise manner (R2/5, R3/4, R1/6, and finally R7), along with non-neuronal cone and pigment cells [46]. Therefore, we also evaluated the effects of Notch overexpression and found no effect on the *KCNC3*^*R423H*^ eye phenotype (S7D and S7H Fig), further supporting specificity for the interaction of the mutant potassium channel allele with Egfr. The connection with EGFR was further corroborated with experiments in the wing demonstrating that co-expression of Egfr-RNAi and *KCNC3*^R423H^ causes more significant abnormalities in wing vein development than either strain alone (Fig 6E–6G). These results thus provide evidence that the developmental effects of *KCNC3*^*R423H*^ expression may be mediated through disruption of EGFR signaling.

### *KCNC3*^*R423H*^ allele contributes to aberrant trafficking of EGFR

**Fig 7:**
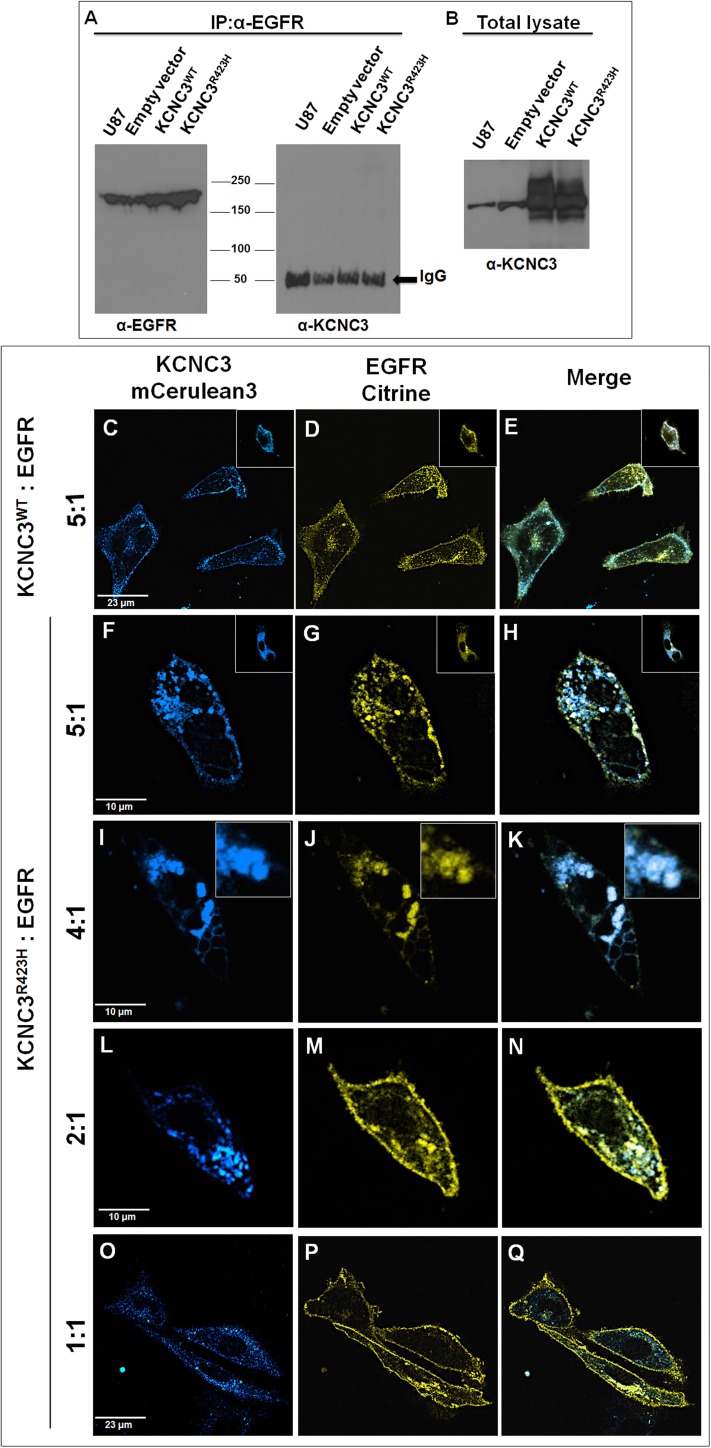
KCNC3^R423H^ causes aberrant EGFR trafficking resulting in intracellular retention. (A) Immunoblot with α-EGFR illustrating positive immunoprecipitation (IP) of EGFR in the eluent from beads bound to anti-EGFR antibody (left panel), with absence of KCNC3 in the same complex (α-KCNC3, right panel). (B) Immunoblot (α-KCNC3) showing presence of KCNC3 in the lysate. (C-E) Confocal fluorescence images of cells co-expressing KCNC3^WT-mCerulean3^:human EGFR^Citrine^ (5:1 ratio), showing membrane localization for both proteins. (F-H) Representative cell co-expressing KCNC3^R423H-mCerulean3^: human EGFR^Citrine^ (5:1 ratio) showing that both proteins do not reach the plasma membrane and are retained in intracellular vesicles. Insets provide additional examples. (I-K) Representative cell co-expressing KCNC3^R423H-mCerulean3^:human EGFR^Citrine^ (4:1 ratio) also showing aberrant trafficking for both proteins. Insets magnify the co-localization of the two proteins in vesicles. (L-N) Representative cell co-expressing KCNC3^R423H-mCerulean3^:human EGFR^Citrine^ (2:1 ratio) showing both plasma membrane trafficking and intracellular retention for EGFR. (O-Q) Confocal fluorescence images of cells co-expressing KCNC3^R423H-mCerulean3^:human EGFR^Citrine^ (1:1 ratio) also demonstrating membrane and intracellular trafficking for EGFR with continued intracellular retention for KCNC3^R423H^. The total amount of DNA used in the co-transfection experiments was kept constant across ratios by adding control plasmid pcDNA 3.1.

To test whether the potassium channel directly interacts with EGFR in mammalian cells, we transiently transfected U87 cells, known to express EGFR [50], with either human *KCNC3*^*WT*^ or *KCNC3*^R423H^.Cellular lysates were subjected to immunoprecipitation with EGFR-specific antibodies. As expected, U87 cells expressed EGFR (Fig 7A, left panel), while immunoblot analyses of the identical immunoprecipitation lysates showed no detectable bands in either the *KCNC3*^*WT*^ or *KCNC3*^*R423H*^ transfected cells (Fig 7A, right panel), despite clear overexpression of KCNC3 (Fig 7B).

With no apparent direct protein-protein interaction, we examined the potential influence of aberrant KCNC3^R423H^ cellular trafficking on EGFR transit through the ER/Golgi to the plasma membrane. Cells were co-transfected with *KCNC3*^*WT-mCerulean3*^ or *KCNC3*^*R423H-mCerulean3*^ and with varying concentrations of a Citrine-tagged human *EGFR* [51] construct, using *KCNC3*:*EGFR* molar ratios from 5:1 to 1:1 (Fig 7C–7Q). Representative confocal Z-stacks of cells expressing both KCNC3^WT^ and EGFR (Fig 7C–7E) illustrate that, despite an excess of *KCNC3*^*WT*^ (5:1), both proteins trafficked normally to the plasma membrane. Conversely, co-expression of KCNC3^R423H^ and EGFR at ratios of 5:1 (Fig 7F–7H) and 4:1 (Fig 7I–7K) reproducibly led to aberrant trafficking of EGFR with sequestration in intracellular vesicles that co-register with KCNC3^R423H^ (Fig 7H and 7K). As the molar ratio of *KCNC3*^*R423H*^:*EGFR* is decreased to 2:1 (Fig 7L–7N), EGFR is found both intracellularly sequestered and at the plasma membrane. At a ratio of 1:1, EGFR traffics normally to the plasma membrane, while in some cells the receptor continues to intracellularly co-register with KCNC3^R423H^ (Fig 7O–7Q). The titration was repeated with KCNC3^R423H^ tagged with a different fluorescent protein, mRuby2, co-expressed with EGFR^Citrine^ at ratios of 3:1 (S8 Fig). These results further illustrate that complete normal trafficking of the receptor to the plasma membrane is achieved only with reduction of the molar ratio of KCNC3^R423H^:EGFR to 0.7:1. To determine whether the mutant channel could cause aberrant trafficking of other membrane proteins, we co-expressed KCNC3^WT^ or KCNC3^R423H^ with N-cadherin (Cadherin-2 [CDH2]) fused with eGFP [52] at molar ratios of 3.5:1 and 2.5:1, with no effects on plasma membrane localization for cadherin (S9 Fig). Together with the rescue of the *Drosophila* eye phenotype (Fig 6E and 6G), these observations strongly implicate a specific link between this causative mutant allele and EGFR.

## Discussion

Previous studies [43, 53, 54] identified KCNC3^R423H^ by screening index ataxia patients from US [15] and European [14, 16] DNA repositories. Our analysis of four pedigrees (3 US, 1 Swedish) unequivocally demonstrates SCA13 causation for this allele. The unifying endophenotype includes infantile onset, non-progressive cerebellar hypoplasia, lifetime improvement of motor and cognitive function, bradyphrenia, dysarthria, tremor, and the classic SCA feature of limb, truncal, and gait ataxia (S3 and S4 Tables). A striking clinical observation is the time- and activity-dependent improvement in motor and cognitive function, despite severe congenital cerebellar hypoplasia (Fig 1).

To address this unique clinical pathology, we have developed fly and cellular models to investigate the underlying developmental, cellular, and biochemical events illuminating mechanisms of neurodevelopmental onset and dominant inheritance. Without SCA13 autopsy specimens, our current understanding of human pathology derives from clinical history and MRI-based cerebellar hypoplasia.

*Drosophila* eye differentiation has previously been used to model alleles causative in SCAs [23, 55–58]. Consistent with neurodegeneration, all these mutant alleles display large, disorganized eyes or a “rough eye” phenotype. In contrast, overexpression of human KCNC3^R423H^ in adult flies results in small, maldeveloped eyes exhibiting fused and disorganized ommatidia and disordered eye bristles, along with aberrant wing veins and shrunken wing formation, which suggests a neurodevelopmental effect. Consistent with a congenital phenotype, expression of KCNC3^R423H^ in third instar larvae caused disturbed patterning and fusion of ommatidial clusters, axonal bundle thinning, and reduced photoreceptor cell clusters. Collectively, these data support the neurodevelopmental nature of KCNC3^R423H^ in SCA13 patients.

Normal trafficking of plasma membrane–targeted proteins involves glycosylation and folding in the ER compartment believed to be followed by vesicular transport to the Golgi for glycan trimming and protein sorting [59]. Vesicles originating at the trans-Golgi network are involved in anterograde transport to the plasma membrane through close association with microtubules and actin filaments [60–62]. Our data show that KCNC3^R423H^ expressed in cell culture is abnormally glycosylated and does not reach the plasma membrane. Co-localization results demonstrate that KCNC3^R423H^ is sequestered in the intravesicular space of either anterograde or endosomal vesicles based on striking co-expression with PI4K2A, a vesicular membrane marker. Consistent with this intracellular sequestration are biophysical studies unequivocally demonstrating absent current conductance in cells expressing this mutation. These data provide an explanation for the deleterious effects of the mutation but alone do not address the dominant inheritance.

To explore the dominant phenotype in SCA13 in light of its tetrameric nature, we calculated the probabilities of tetramer formation relative to expressed ratios of KCNC3^WT^ and KCNC3^R423H^ (S4A and S4B Fig). We provide three lines of experimental evidence that KCNC3^R423H^ exerts a strong dominant negative effect on KCNC3^WT^ monomers (Figs 4 and 5). Self-crosses in the fly eye and wing show that KCNC3^WT^ does not overcome the effects of the mutant allele, along with co-expression in cell culture, where at a ratio as high as 6:1 (WT:R423H), KCNC3^WT^ remains mis-trafficked and intracellularly localized. Functionally, these data are further supported with biophysical data showing that <20% current conductance is detectable at a 1:1 ratio of WT:R423H. Collectively, these data form a strong basis for dominant inheritance but do not address the neurodevelopmental phenotype.

The Egfr and Notch morphogenetic pathways are prominent pathways involved in *Drosophila* eye and wing development [46, 63, 64]. dEgfr is required for recruitment of neuronal and non-neuronal cells in the ommatidium [29, 65]. Because the profound KCNC3^R423H^ eye phenotype can be almost completely rescued by elevated Egfr expression and R423H/Egfr-RNAi co-expression accentuates wing malformations, a direct or indirect association is strongly implicated between KCNC3 and Egfr, as well as a link between this potent growth factor receptor and cerebellar hypoplasia. Consistent with the fly data, co-expression of EGFR with KCNC3^R423H^ in mammalian cells leads to aberrant intracellular retention of EGFR that co-registers in vesicles with the mutant channel, the same anterograde and/or endosomal vesicles positive for PI4K2A. Normal EGFR plasma membrane localization is overcome only with a decrease in the concentration of the mutant channel below a ratio of 1:1. Negative co-immunoprecipitation data confirms that the intracellular retention of EGFR by KCNC3^R423H^ is not likely to be dependent on a direct protein-to-protein interactions. Therefore, our current hypothesis involves the intracellular sequestration of EGFR through yet unknown effects of KCNC3^R423H^ in anterograde or endosomal vesicles during cerebellar development. This hypothesis can be rationalized in part by previous studies in rats proving the presence of EGFR in Purkinje cells during late cerebellar development [66, 67]. It is also relevant to note that PI4K2A has been shown to co-localize with protein markers of the late endosome and is required for endocytic trafficking and degradation and/or downregulation of EGFR [68]. Future studies to address the interplay of KCNC3^R423H^, EGFR, and PI4K2A will provide important insights into the mechanisms governing normal cerebellar development and cerebellar hypoplasia in SCA13.

## Supporting information

S1 Table Cloning strategies. (TIF)

S2 Table Driver and responder Drosophila lines. (TIF)

S3 Table Summary of phenotypic data derived from patient clinical history. (TIF)

S4 Table Summary of ataxic parameters derived from patient clinical history. (TIF)

S5 Table Electrophysiology data. (TIF)

S1 Fig Drosophila and cell expression studies. (TIF)

S2 Fig Expression of KCNC3^R423H^ in Drosophila adult eyes and larval eye discs. (TIF)

S3 Fig Fluorescence and bright field microscopy of CHO cells transiently expressing human KCNC3^WT^ or KCNC3^R423H^ as C-terminal fusion proteins with the GFP derivative, mCerulean3. (TIF)

S4 Fig Tetramer formation possibilities and probabilities. (TIF)

S5 Fig Effect of EgfrRNAi co-expression with KCNC3^R423H^ in Drosophila. (TIF)

S6 Fig Drosophila and Mammalian EGFR pathways. (TIF)

S7 Fig Expression of downstream factors of the Egfr signaling pathway and Notch, with KCNC3^R423H^. (TIF)

S8 Fig Co-expression of KCNC3^R423H-mRuby2^ and EGFR^Citrine^. (TIF)

S9 Fig Effect of KCNC3^R423H^ on Cadherin trafficking. (TIF)

## References

[1] Matilla-Dueñas A. The ever expanding spinocerebellar ataxias. Editorial. Cerebellum. 2012;11:821–7. doi: 10.1007/s12311-012-0376-4 22447528 10.1007/s12311-012-0376-4

[2] Orr HT. Cell biology of spinocerebellar ataxia. The Journal of Cell Biology. 2012;197:167–77. doi: 10.1083/jcb.201105092 22508507 10.1083/jcb.201105092 PMC3328388

[3] Seidel K, Siswanto S, Brunt ERP, Den Dunnen W, Korf HW, Rüb U. Brain pathology of spinocerebellar ataxias. Acta Neuropathologica 2012 p. 1–21. 10.1007/s00401-012-1000-x 22684686

[4] Jayadev S, Bird TD. Hereditary ataxias: overview. Genetics in medicine: official journal of the American College of Medical Genetics. 2013;15:673–83. 23538602 10.1038/gim.2013.28

[5] Matilla-Dueñas A, Ashizawa T, Brice A, Magri S, McFarland KN, Pandolfo M, et al. Consensus paper: Pathological mechanisms underlying neurodegeneration in spinocerebellar ataxias2014. 10.1007/s12311-013-0539-y PMC3943639 24307138

[6] Waters MF, Fee D, Figueroa KP, Nolte D, Müller U, Advincula J, et al An autosomal dominant ataxia maps to 19q13: Allelic heterogeneity of SCA13 or novel locus? Neurology. 2005;65:1111–3. doi: 10.1212/01.wnl.0000177490.05162.41 16135769 10.1212/01.wnl.0000177490.05162.41

[7] Waters MF, Minassian Na, Stevanin G, Figueroa KP, Bannister JPa, Nolte D, et al Mutations in voltage-gated potassium channel KCNC3 cause degenerative and developmental central nervous system phenotypes. Nature genetics. 2006;38:447–51. doi: 10.1038/ng1758 16501573 10.1038/ng1758

[8] Subramony SH, Advincula J, Perlman S, Rosales RL, Lee LV, Ashizawa T, et al Comprehensive phenotype of the p.arg420his allelic form of spinocerebellar ataxia type 13. Cerebellum. 2013;12:932–6. doi: 10.1007/s12311-013-0507-6 23912307 10.1007/s12311-013-0507-6 PMC3824261

[9] Chang SY, Zagha E, Kwon ES, Ozaita A, Bobik M, Martone ME, et al Distribution of Kv3. 3 potassium channel subunits in distinct neuronal populations of mouse brain. Journal of Comparative Neurology. 2007;502:953–72. doi: 10.1002/cne.21353 17444489 10.1002/cne.21353

[10] Zagha E, Manita S, Ross WN, Rudy B. Dendritic Kv3.3 potassium channels in cerebellar purkinje cells regulate generation and spatial dynamics of dendritic Ca2+ spikes. Journal of neurophysiology. 2010;103:3516–25. doi: 10.1152/jn.00982.2009 20357073 10.1152/jn.00982.2009 PMC2888543

[11] Jensen MØ, Jogini V, Borhani DaW, Leffler AE, Dror RO, Shaw DE. Mechanism of voltage gating in potassium channels. Science. 2012;336:229–33. doi: 10.1126/science.1216533 22499946 10.1126/science.1216533

[12] Mechanisms of activation of voltage-gated potassium channels, (2014). PMC4273088 25558391

[13] Herman-Bert a, Stevanin G, Netter JC, Rascol O, Brassat D, Calvas P, et al Mapping of spinocerebellar ataxia 13 to chromosome 19q13.3-q13.4 in a family with autosomal dominant cerebellar ataxia and mental retardation. American journal of human genetics. 2000;67:229–35. 10820125 10.1086/302958 PMC1287081

[14] Figueroa KP, Minassian NA, Stevanin G, Waters M, Garibyan V, Forlani S, et al KCNC3: Phenotype, mutations, channel biophysics—A study of 260 familial ataxia patients. Human Mutation. 2010;31:191–6. doi: 10.1002/humu.21165 19953606 10.1002/humu.21165 PMC2814913

[15] Figueroa KP, Waters MF, Garibyan V, Bird TD, Gomez CM, Ranum LPW, et al Frequency of KCNC3 DNA variants as causes of spinocerebellar ataxia 13 (SCA13). PLoS ONE. 2011;6. 10.1371/journal.pone.0017811 PMC3066194 21479265

[16] Duarri A, Nibbeling EAR, Fokkens MR, Meijer M, Boerrigter M, Verschuuren-Bemelmans CC, et al Functional analysis helps to define KCNC3 mutational spectrum in Dutch ataxia cases. PLoS ONE. 2015;10. 10.1371/journal.pone.0116599 PMC4355074 25756792

[17] Lam AJ, St-Pierre F, Gong Y, Marshall JD, Cranfill PJ, Baird Ma, et al Improving FRET dynamic range with bright green and red fluorescent proteins. Nature methods. 2012;9:1005–12. doi: 10.1038/nmeth.2171 22961245 10.1038/nmeth.2171 PMC3461113

[18] Markwardt ML, Kremers GJ, Kraft CA, Ray K, Cranfill PJC, Wilson KA, et al An improved cerulean fluorescent protein with enhanced brightness and reduced reversible photoswitching. PLoS ONE. 2011;6. 10.1371/journal.pone.0017896 PMC3066204 21479270

[19] Gallego-Iradi C, Bickford JS, Khare S, Hall A, Nick JA, Salmasinia D, et al KCNC3R420H, a K+ channel mutation causative in spinocerebellar ataxia 13 displays aberrant intracellular trafficking. Neurobiology of Disease. 2014;71:270–9. doi: 10.1016/j.nbd.2014.08.020 25152487 10.1016/j.nbd.2014.08.020 PMC4181561

[20] Singh A, Kango-Singh M, Sun YH. Eye suppression, a novel function of teashirt, requires Wingless signaling. Development (Cambridge, England). 2002;129:4271–80. 10.1242/dev.129.18.4271 12183379

[21] Kim BR, Lim JH, Lee SA, Park S, Koh SE, Lee IS, et al Usefulness of the Scale for the Assessment and Rating of Ataxia (SARA) in Ataxic Stroke Patients. Ann Rehabil Med. 2011;35(6):772–80. PubMed Central PMCID: PMCPMC3309386. doi: 10.5535/arm.2011.35.6.772 22506205 10.5535/arm.2011.35.6.772 PMC3309386

[22] Bauer P, Stevanin G, Beetz C, Synofzik M, Schmitz-Hübsch T, Wüllner U, et al Spinocerebellar ataxia type 11 (SCA11) is an uncommon cause of dominant ataxia among French and German kindreds. Journal of neurology, neurosurgery, and psychiatry. 2010;81:1229–32. doi: 10.1136/jnnp.2009.202150 20667868 10.1136/jnnp.2009.202150

[23] Lorenzo DN, Li MG, Mische SE, Armbrust KR, Ronum LPW, Hays TS. Spectrin mutations that cause spinocerebellar ataxia type 5 impair axonal transport and induce neurodegeneration in Dmsophila. Journal of Cell Biology. 2010;189:143–58. doi: 10.1083/jcb.200905158 20368622 10.1083/jcb.200905158 PMC2854382

[24] Marelli C, van de Leemput J, Johnson JO, Tison F, Thauvin-Robinet C, Picard F, et al SCA15 due to large ITPR1 deletions in a cohort of 333 white families with dominant ataxia. Archives of neurology. 2011;68:637–43. doi: 10.1001/archneurol.2011.81 21555639 10.1001/archneurol.2011.81 PMC3142680

[25] Brand AH, Perrimon N. Targeted gene expression as a means of altering cell fates and generating dominant phenotypes. Development (Cambridge, England). 1993;118:401–15. 10.1242/dev.118.2.401 8223268

[26] Smith JE, Cronmiller C. The Drosophila daughterless gene autoregulates and is controlled by both positive and negative cis regulation. Development (Cambridge, England). 2001;128:4705–14. 10.1242/dev.128.23.4705 11731451

[27] Duffy JB. GAL4 system in Drosophila: a fly geneticist's Swiss army knife. Genesis. 2002;34(1–2):1–15. doi: 10.1002/gene.10150 12324939 10.1002/gene.10150

[28] Haerry TE, Khalsa O, O'Connor MB, Wharton KA. Synergistic signaling by two BMP ligands through the SAX and TKV receptors controls wing growth and patterning in Drosophila. Development. 1998;125:3977–87. 9735359 10.1242/dev.125.20.3977

[29] Freeman M. Cell determination strategies in the Drosophila eye. Development (Cambridge, England). 1997;124:261–70. 10.1242/dev.124.2.261 9053303

[30] Gene elav of Drosophila melanogaster: A prototype for neuronal-specific RNA binding protein gene family that is conserved in flies and humans, (1993). 10.1002/neu.480240604 8331337

[31] Reinke R, Krantz DE, Yen D, Lawrence Zipursky S. Chaoptin, a cell surface glycoprotein required for Drosophila photoreceptor cell morphogenesis, contains a repeat motif found in yeast and human. Cell. 1988;52:291–301. 3124963 10.1016/0092-8674(88)90518-1

[32] Waters MF, Pulst SM. SCA13. Cerebellum. 2008;7:165–9. doi: 10.1007/s12311-008-0039-7 18592334 10.1007/s12311-008-0039-7

[33] Sigma-1 receptor: The novel intracellular target of neuropsychotherapeutic drugs, (2015). 10.1016/j.jphs.2014.07.001 25704011

[34] Zuchero JB, Coutts AS, Quinlan ME, Thangue NBL, Mullins RD. p53-cofactor JMY is a multifunctional actin nucleation factor. Nature cell biology. 2009;11:451–9. doi: 10.1038/ncb1852 19287377 10.1038/ncb1852 PMC2763628

[35] Coutts AS, Weston L, La Thangue NB. A transcription co-factor integrates cell adhesion and motility with the p53 response. Proceedings of the National Academy of Sciences of the United States of America. 2009;106:19872–7. doi: 10.1073/pnas.0906785106 19897726 10.1073/pnas.0906785106 PMC2785259

[36] Schlüter K, Waschbüsch D, Anft M, Hügging D, Kind S, Hänisch J, et al JMY is involved in anterograde vesicle trafficking from the trans-Golgi network. European Journal of Cell Biology. 2014;93:194–204. doi: 10.1016/j.ejcb.2014.06.001 25015719 10.1016/j.ejcb.2014.06.001

[37] Wang YJ, Wang J, Sun HQ, Martinez M, Sun YX, Macia E, et al Phosphatidylinositol 4 phosphate regulates targeting of clathrin adaptor AP-1 complexes to the Golgi. Cell. 2003;114:299–310. 12914695 10.1016/s0092-8674(03)00603-2

[38] Craige B, Salazar G, Faundez V. Phosphatidylinositol-4-Kinase Type II Alpha Contains an AP-3–sorting Motif and a Kinase Domain That Are Both Required for Endosome Traffic. Molecular biology of the cell. 2008;19:1415–26. doi: 10.1091/mbc.E07-12-1239 18256276 10.1091/mbc.E07-12-1239 PMC2291421

[39] Mammalian phosphatidylinositol 4-kinases as modulators of membrane trafficking and lipid signaling networks, (2013). 10.1016/j.plipres.2013.04.002 PMC3989048 23608234

[40] Rudy B, McBain CJ. Kv3 channels: Voltage-gated K+ channels designed for high-frequency repetitive firing. Trends in Neurosciences. 2001;24:517–26. 11506885 10.1016/s0166-2236(00)01892-0

[41] Hurlock EC, McMahon A, Joho RH. Purkinje-cell-restricted restoration of Kv3.3 function restores complex spikes and rescues motor coordination in Kcnc3 mutants. The Journal of neuroscience: the official journal of the Society for Neuroscience. 2008;28:4640–8. 18448641 10.1523/JNEUROSCI.5486-07.2008 PMC6670432

[42] Zhang Y, Kaczmarek LK. Kv3.3 potassium channels and spinocerebellar ataxia. The Journal of Physiology. 2015;00:1–8. 10.1113/JP271343 PMC4983625 26442672

[43] Minassian Na, Lin M-Ca, Papazian DM. Altered Kv3.3 channel gating in early-onset spinocerebellar ataxia type 13. The Journal of physiology. 2012;590:1599–614. doi: 10.1113/jphysiol.2012.228205 22289912 10.1113/jphysiol.2012.228205 PMC3413486

[44] Kurada P, White K. Epidermal growth factor receptor: its role in Drosophila eye differentiation and cell survival. Apoptosis: an international journal on programmed cell death. 1999;4:239–43. 14634274 10.1023/a:1009648724937

[45] Nagaraj R, Banerjee U. The little R cell that could. International Journal of Developmental Biology 2004 p. 755–60. doi: 10.1387/ijdb.041881rn 15558468 10.1387/ijdb.041881rn

[46] Doroquez DB, Rebay I. Signal integration during development: mechanisms of EGFR and Notch pathway function and cross-talk. Critical reviews in biochemistry and molecular biology. 2006;41:339–85. doi: 10.1080/10409230600914344 17092823 10.1080/10409230600914344

[47] Guichard A, Srinivasan S, Zimm G, Bier E. A screen for dominant mutations applied to components in the Drosophila EGF-R pathway. Proceedings of the National Academy of Sciences of the United States of America. 2002;99:3752–7. doi: 10.1073/pnas.052028699 11904431 10.1073/pnas.052028699 PMC122596

[48] Wolff T, Ready DF. The beginning of pattern formation in the Drosophila compound eye: the morphogenetic furrow and the second mitotic wave. Development (Cambridge, England). 1991;113:841–50. 10.1242/dev.113.3.841 1726564

[49] Roignant J-Y, Treisman JE. Pattern formation in the Drosophila eye disc. The International journal of developmental biology. 2009;53:795–804. doi: 10.1387/ijdb.072483jr 19557685 10.1387/ijdb.072483jr PMC2713679

[50] Maity A, Pore N, Lee J, Solomon D, O'Rourke DM. Epidermal growth factor receptor transcriptionally up-regulates vascular endothelial growth factor expression in human glioblastoma cells via a pathway involving phosphatidylinositol 3'-kinase and distinct from that induced by hypoxia. Cancer Research. 2000;60:5879–86. 11059786

[51] Offterdinger M, Bastiaens PI. Prolonged EGFR signaling by ERBB2-mediated sequestration at the plasma membrane. Traffic. 2008;9:147–55. doi: 10.1111/j.1600-0854.2007.00665.x 17956594 10.1111/j.1600-0854.2007.00665.x

[52] Nechiporuk T, Fernandez TE, Vasioukhin V. Failure of epithelial tube maintenance causes hydrocephalus and renal cysts in Dlg5-/- mice. Dev Cell. 2007;13(3):338–50. PubMed Central PMCID: PMCPMC2023971. doi: 10.1016/j.devcel.2007.07.017 17765678 10.1016/j.devcel.2007.07.017 PMC2023971

[53] Zhao J, Zhu J, Thornhill WB. Spinocerebellar ataxia-13 Kv3.3 potassium channels: arginine-to-histidine mutations affect both functional and protein expression on the cell surface. The Biochemical journal. 2013;454:259–65. doi: 10.1042/BJ20130034 23734863 10.1042/BJ20130034

[54] Irie T, Matsuzaki Y, Sekino Y, Hirai H. Kv3.3 channels harbouring a mutation of spinocerebellar ataxia type 13 alter excitability and induce cell death in cultured cerebellar Purkinje cells. The Journal of physiology. 2014;592:229–47. doi: 10.1113/jphysiol.2013.264309 24218544 10.1113/jphysiol.2013.264309 PMC3903362

[55] Fernandez-Funez P, Nino-Rosales ML, de Gouyon B, She W-C, Luchak JM, Martinez P, et al Identification of genes that modify ataxin-1-induced neurodegeneration. Nature. 2000;408:101–6. doi: 10.1038/35040584 11081516 10.1038/35040584

[56] Mutsuddi M, Marshall CM, Benzow KA, Koob MD, Rebay I. The Spinocerebellar Ataxia 8 noncoding RNA causes neurodegeneration and associates with Staufen in Drosophila. Current Biology. 2004;14:302–8. doi: 10.1016/j.cub.2004.01.034 14972680 10.1016/j.cub.2004.01.034

[57] Park J, Al-Ramahi I, Tan Q, Mollema N, Diaz-Garcia JR, Gallego-Flores T, et al RAS-MAPK-MSK1 pathway modulates ataxin 1 protein levels and toxicity in SCA1. Nature. 2013;498:325–31. doi: 10.1038/nature12204 23719381 10.1038/nature12204 PMC4020154

[58] Snijder PM, Baratashvili M, Grzeschik NA, Leuvenink HGD, Kuijpers L, Huitema S, et al Overexpression of cystathionine γ-lyase suppresses detrimental effects of spinocerebellar ataxia type 3. Molecular medicine (Cambridge, Mass). 2015;53:160. 10.2119/molmed.2015.00221 PMC4749487 26467707

[59] Brandizzi F, Barlowe C. Organization of the ER-Golgi interface for membrane traffic control. Nature reviews Molecular cell biology. 2013;14:382–92. doi: 10.1038/nrm3588 23698585 10.1038/nrm3588 PMC4064004

[60] Fort AG, Murray JW, Dandachi N, Davidson MW, Dermietzel R, Wolkoff AW, et al In vitro motility of liver connexin vesicles along microtubules utilizes kinesin motors. Journal of Biological Chemistry. 2011;286:22875–85. doi: 10.1074/jbc.M111.219709 21536677 10.1074/jbc.M111.219709 PMC3123055

[61] Smyth JW, Vogan JM, Buch PJ, Zhang SS, Fong TS, Hong TT, et al Actin cytoskeleton rest stops regulate anterograde traffic of connexin 43 vesicles to the plasma membrane. Circulation Research. 2012;110:978–89. doi: 10.1161/CIRCRESAHA.111.257964 22328533 10.1161/CIRCRESAHA.111.257964 PMC3621031

[62] Hancock WO. Bidirectional cargo transport: moving beyond tug of war. Nature Reviews Molecular Cell Biology. 2014;15:615–28. doi: 10.1038/nrm3853 25118718 10.1038/nrm3853 PMC5014371

[63] Doroquez DB, Orr-Weaver TL, Rebay I. Split ends antagonizes the Notch and potentiates the EGFR signaling pathways during Drosophila eye development. Mechanisms of Development. 2007;124:792–806. doi: 10.1016/j.mod.2007.05.002 17588724 10.1016/j.mod.2007.05.002 PMC2231642

[64] Price JV, Savenye ED, Lum D, Breitkreutz A. Dominant enhancers of Egfr in Drosophila melanogaster: Genetic links between the Notch and Egfr signaling pathways. Genetics. 1997;147:1139–53. 9383058 10.1093/genetics/147.3.1139 PMC1208239

[65] Kumar JP. Signalling pathways in Drosophila and vertebrate retinal development. Nature reviews Genetics. 2001;2:846–57. doi: 10.1038/35098564 11715040 10.1038/35098564

[66] Gómez-Pinilla F, Knauer DJ, Nieto-Sampedro M. Epidermal growth factor receptor immunoreactivity in rat brain. Development and cellular localization. Brain research. 1988;438:385–90. 3345447 10.1016/0006-8993(88)91369-8

[67] The neurotrophic action and signalling of epidermal growth factor, (1997). 10.1016/s0301-0082(96)00046-9 9044427

[68] Minogue S, Waugh MG, De Matteis MA, Stephens DJ, Berditchevski F, Hsuan JJ. Phosphatidylinositol 4-kinase is required for endosomal trafficking and degradation of the EGF receptor. Journal of cell science. 2006;119:571–81. doi: 10.1242/jcs.02752 16443754 10.1242/jcs.02752

