# Supplementary figures and images for: A KCNC3 mutation causes a neurodevelopmental, non-progressive SCA13 subtype associated with dominant negative effects and aberrant EGFR trafficking
Source: PLoS One. 2017 May 3;12(5):e0173565. doi: 10.1371/journal.pone.0173565 (PMC5414954; doi:10.1371/journal.pone.0173565)

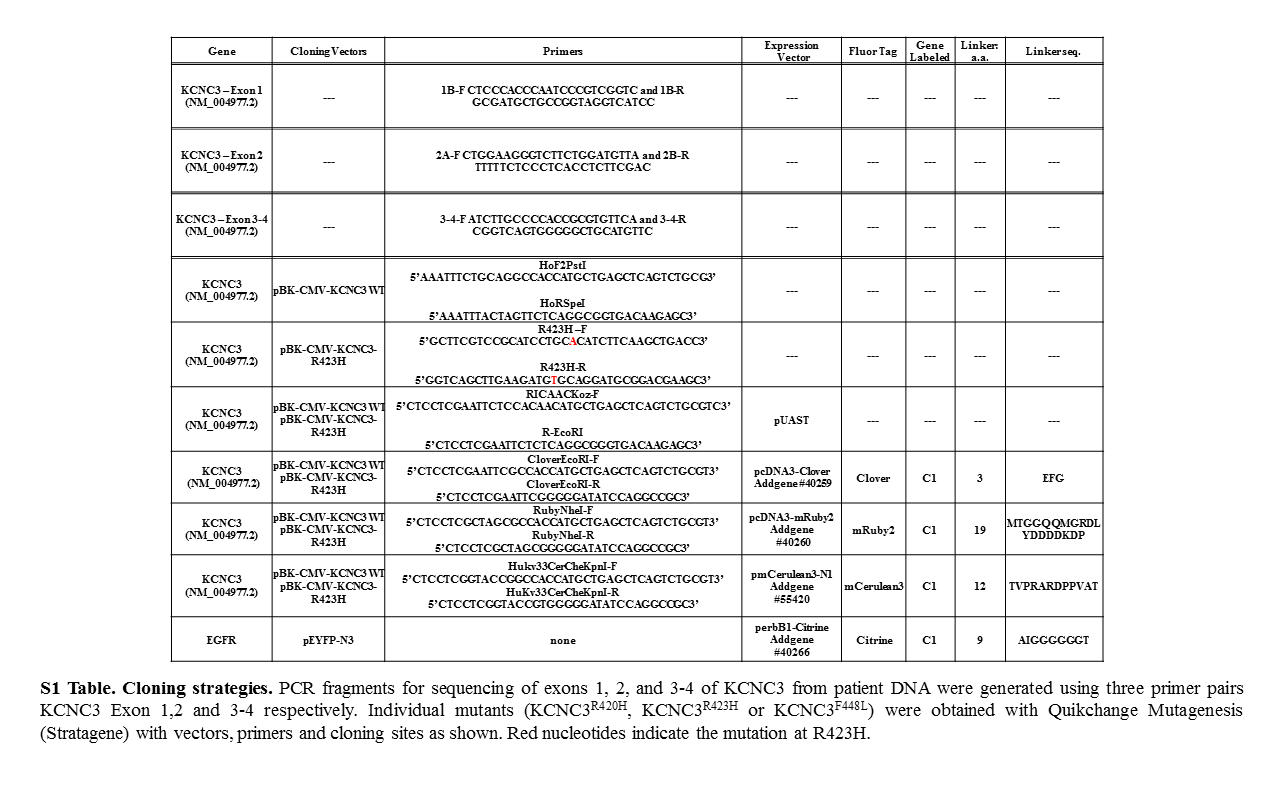

Supplement: S1 Table — (TIF) [file pone.0173565.s001.TIF]

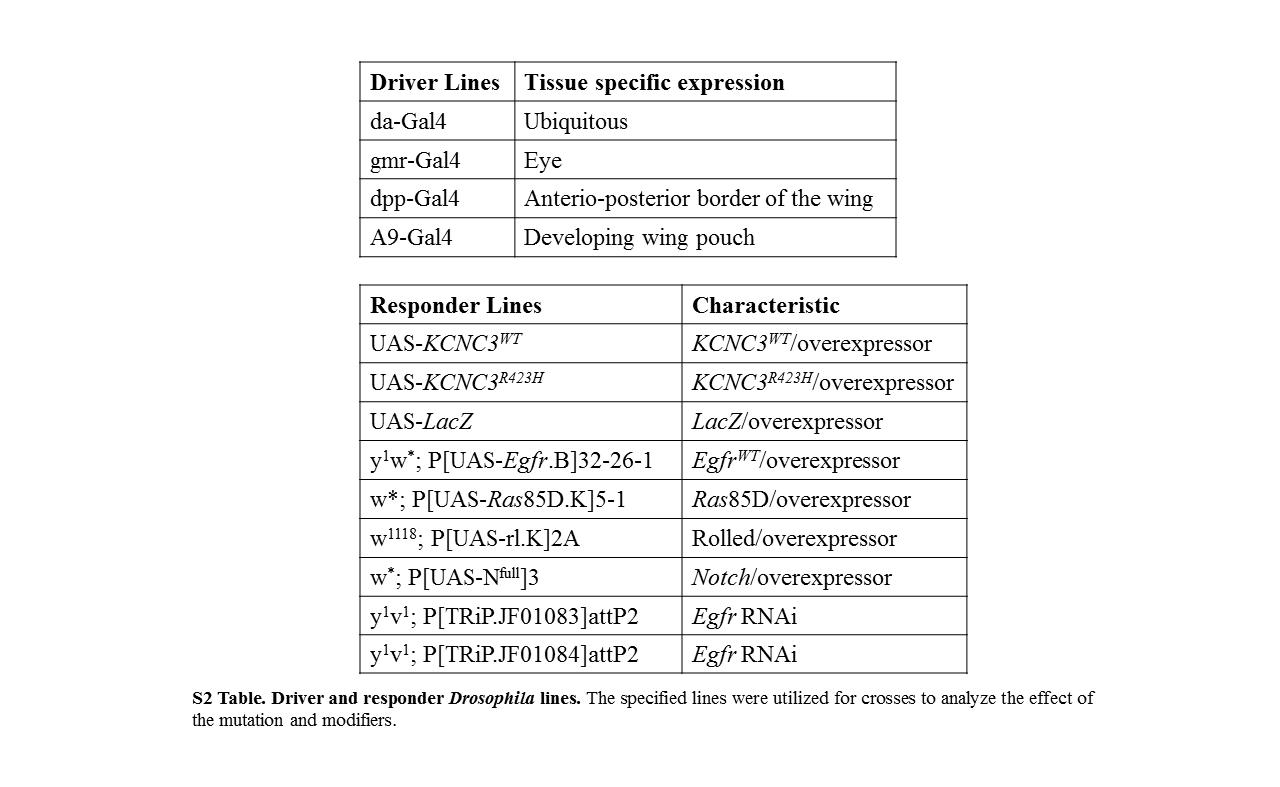

Supplement: S2 Table — (TIF) [file pone.0173565.s002.TIF]

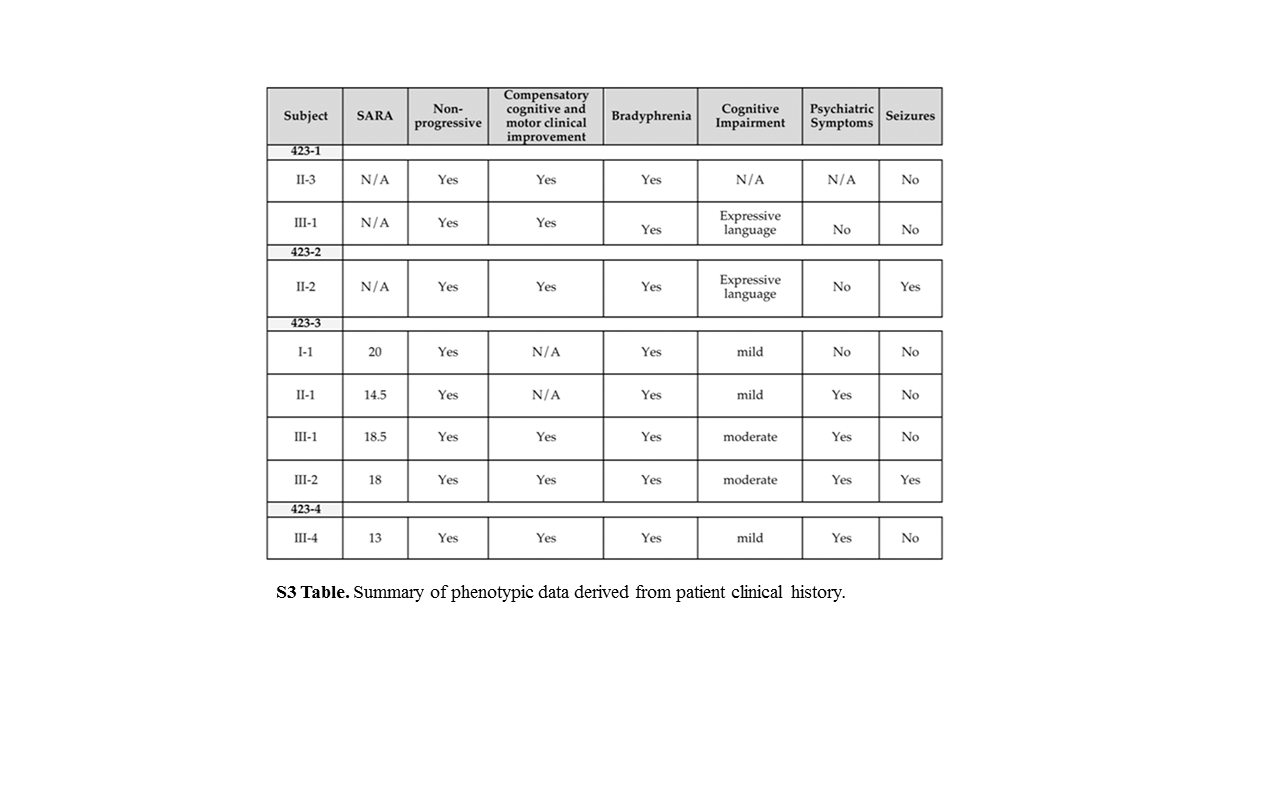

Supplement: S3 Table — (TIF) [file pone.0173565.s003.TIF]

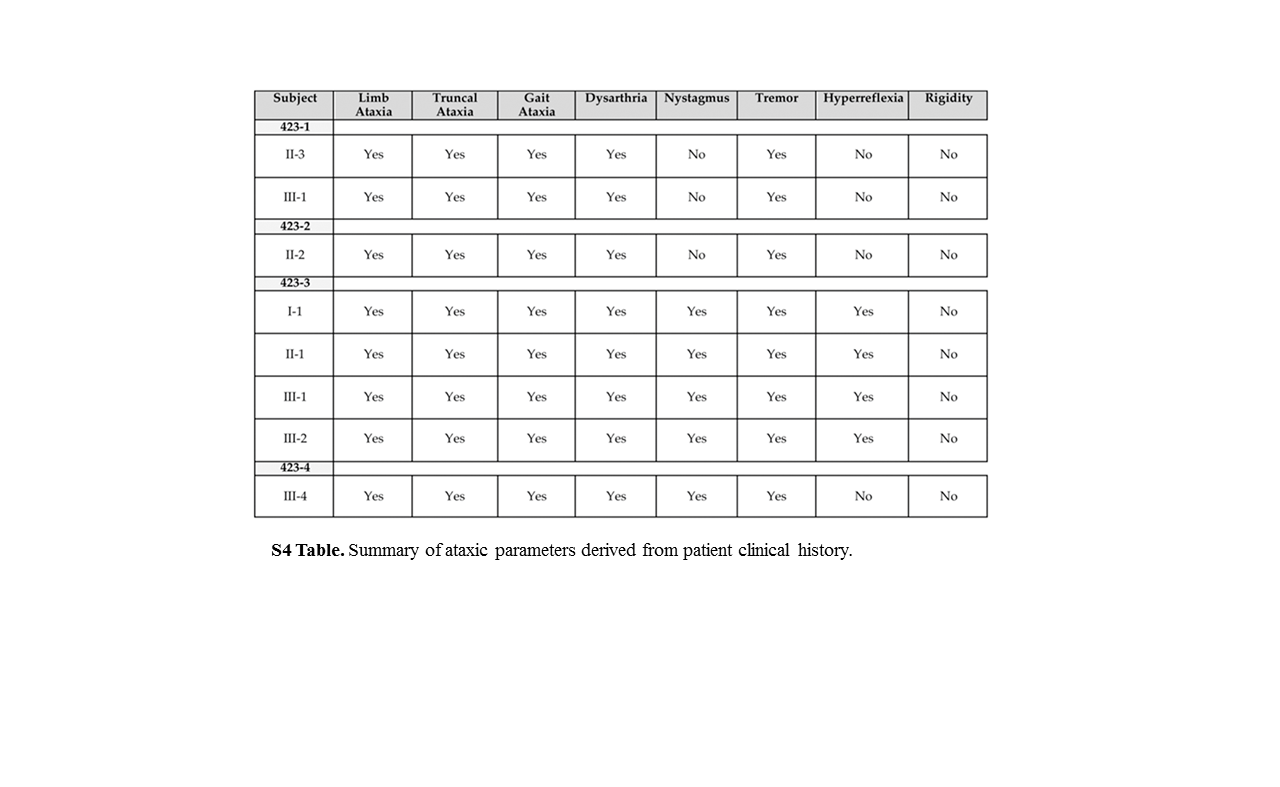

Supplement: S4 Table — (TIF) [file pone.0173565.s004.TIF]

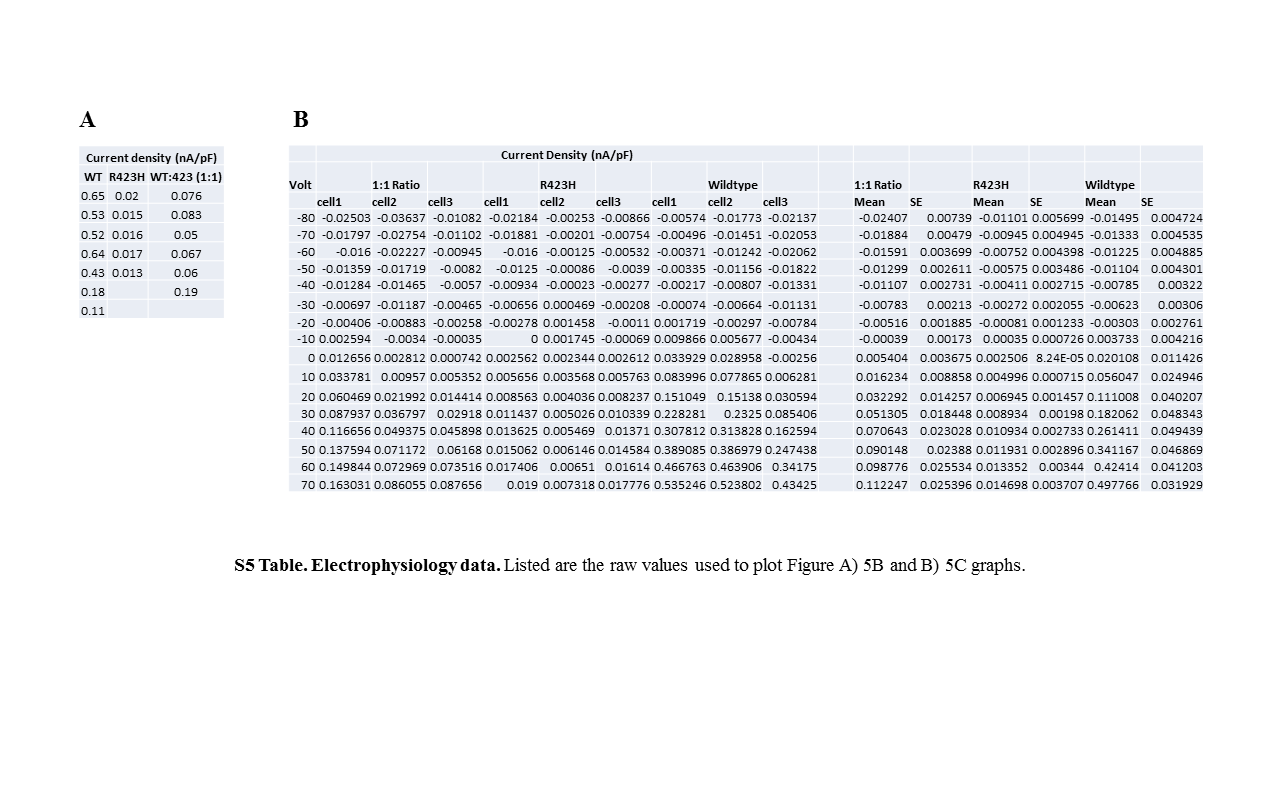

Supplement: S5 Table — (TIF) [file pone.0173565.s005.TIF]

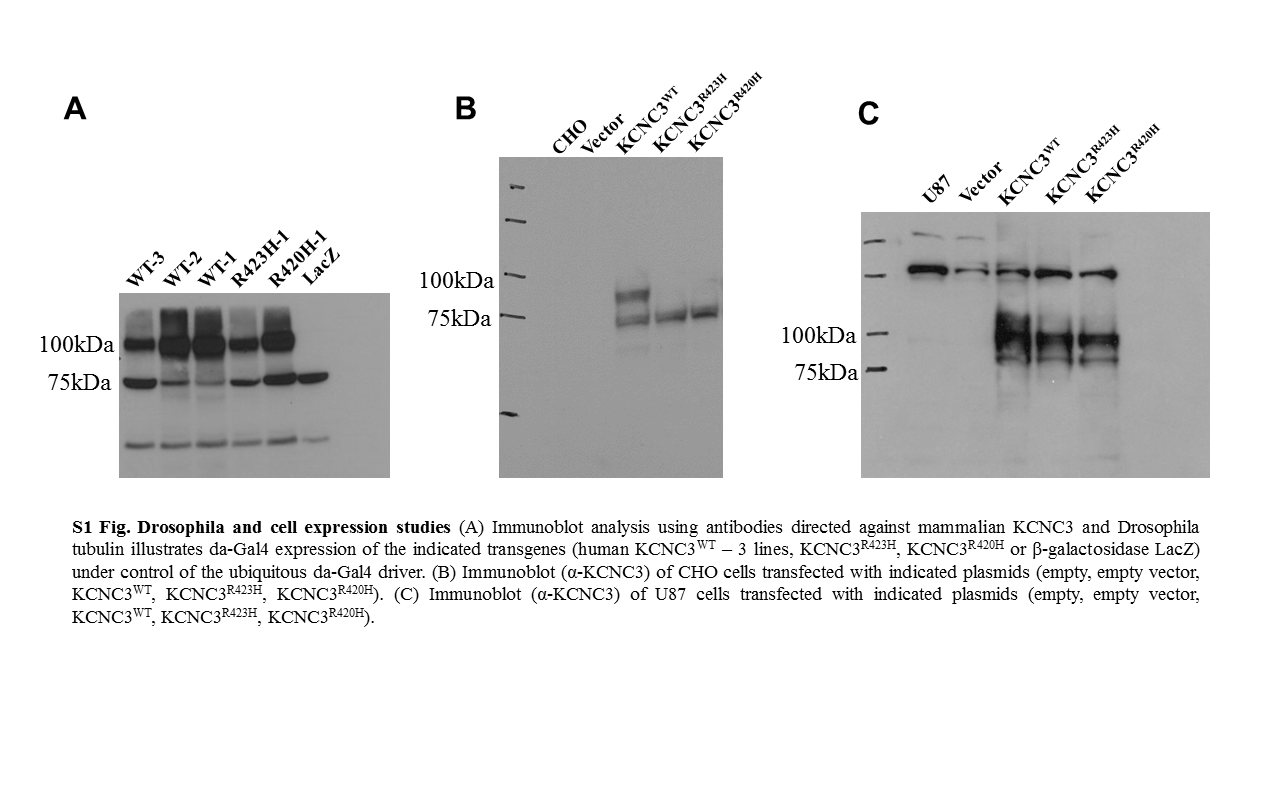

Supplement: S1 Fig — (TIF) [file pone.0173565.s006.TIF]

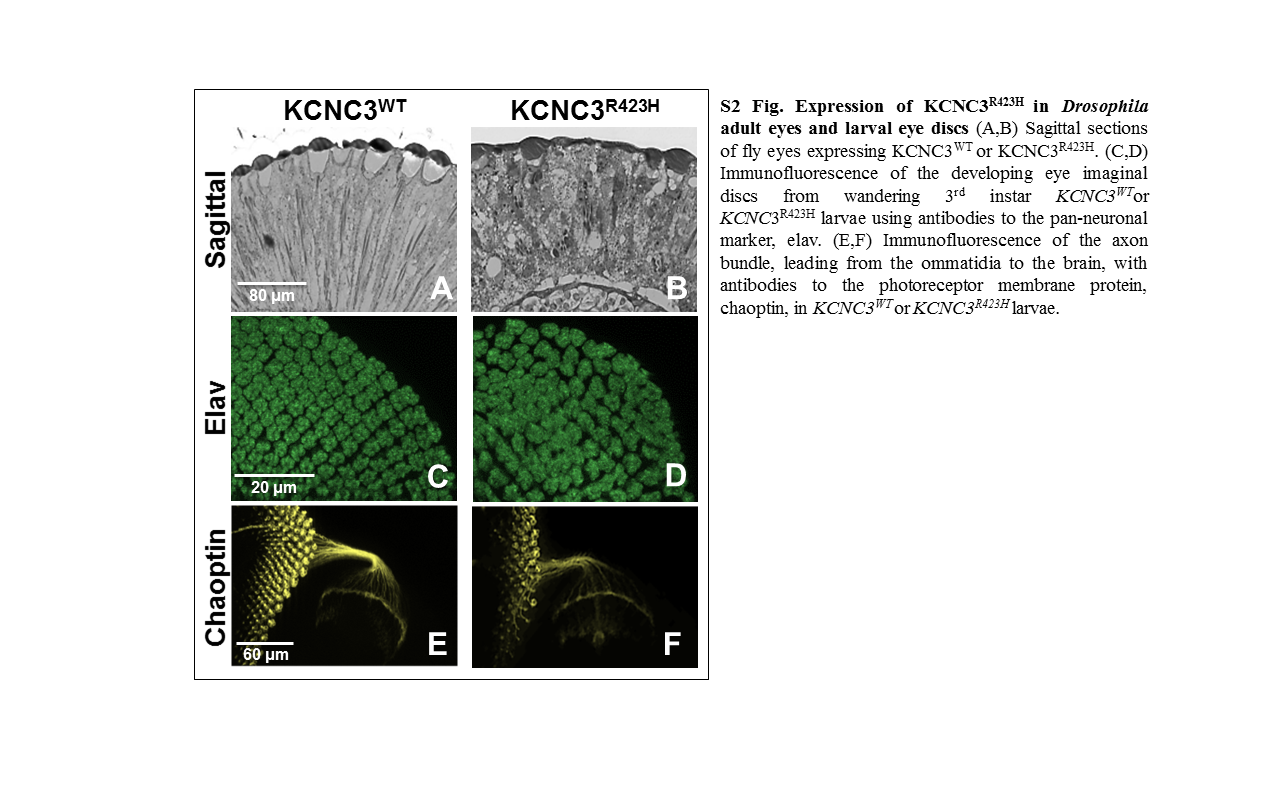

Supplement: S2 Fig — (TIF) [file pone.0173565.s007.TIF]

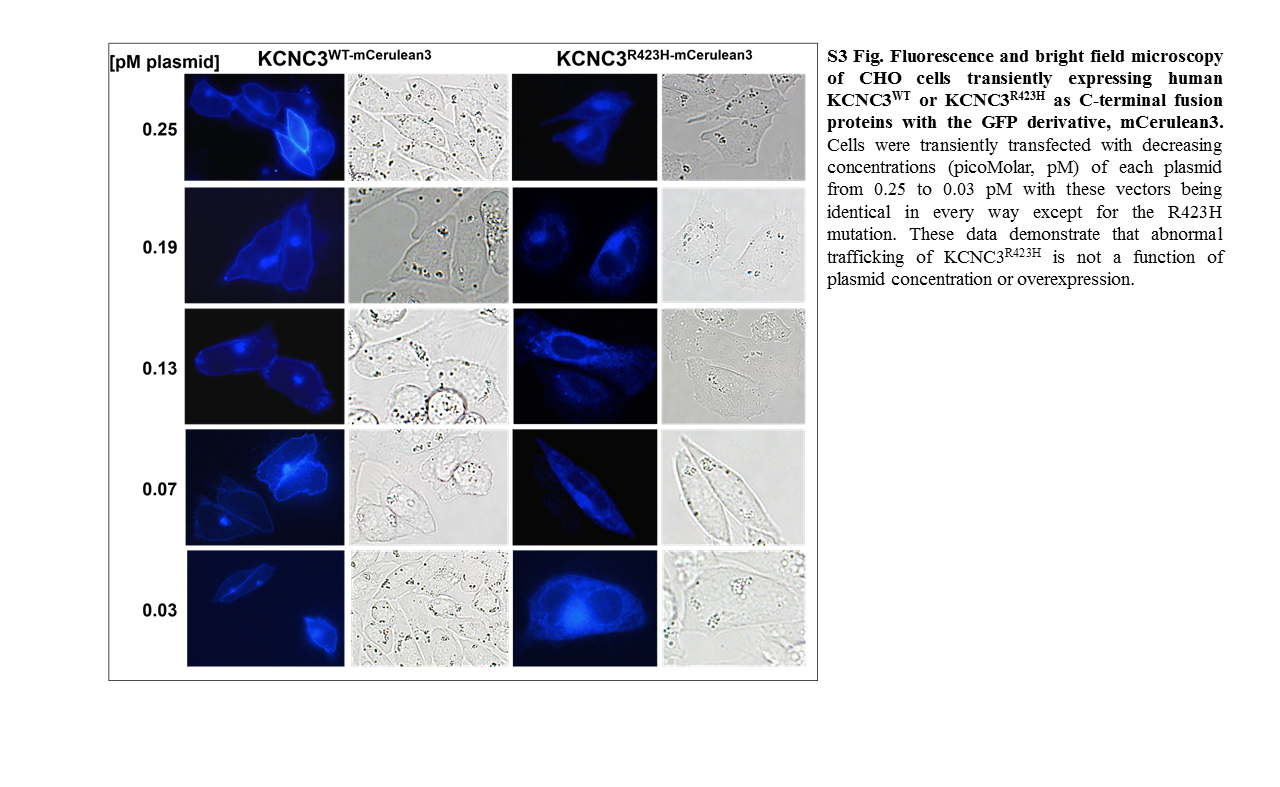

Supplement: S3 Fig — (TIF) [file pone.0173565.s008.TIF]

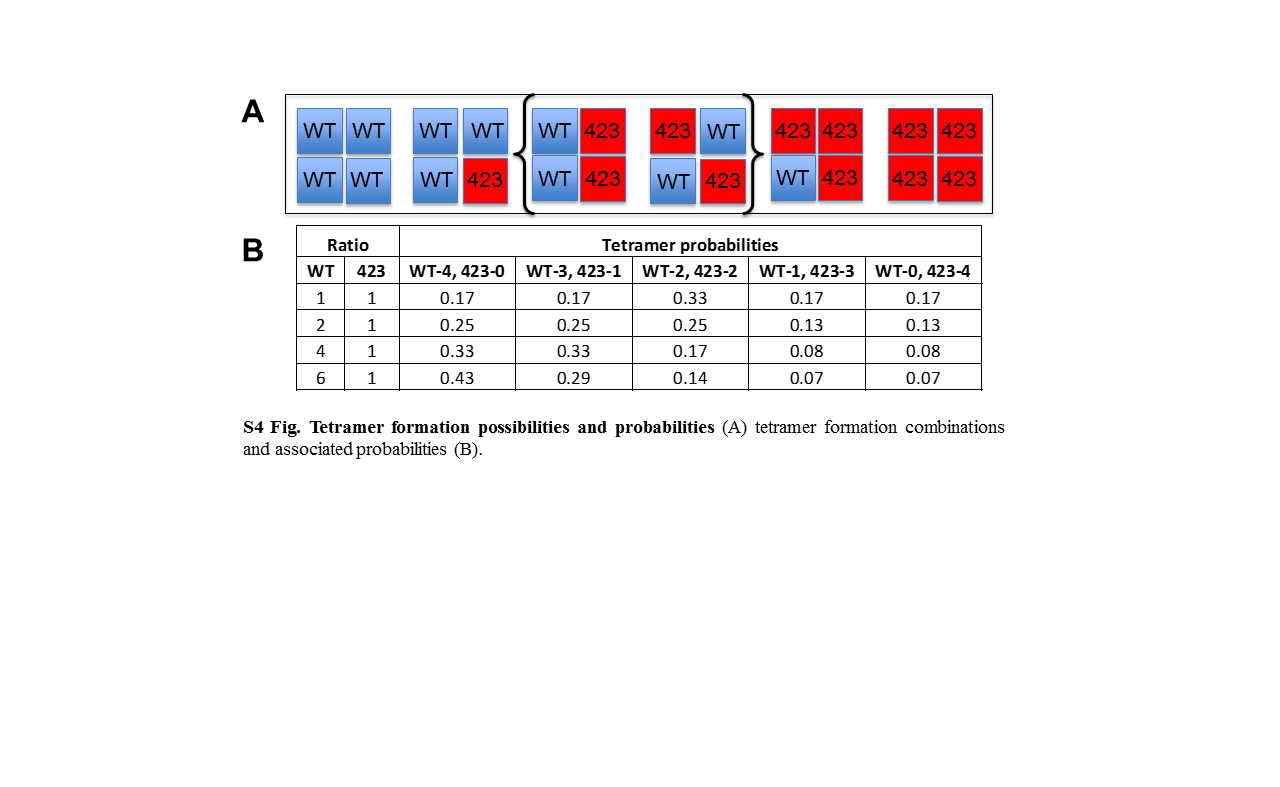

Supplement: S4 Fig — (TIF) [file pone.0173565.s009.TIF]

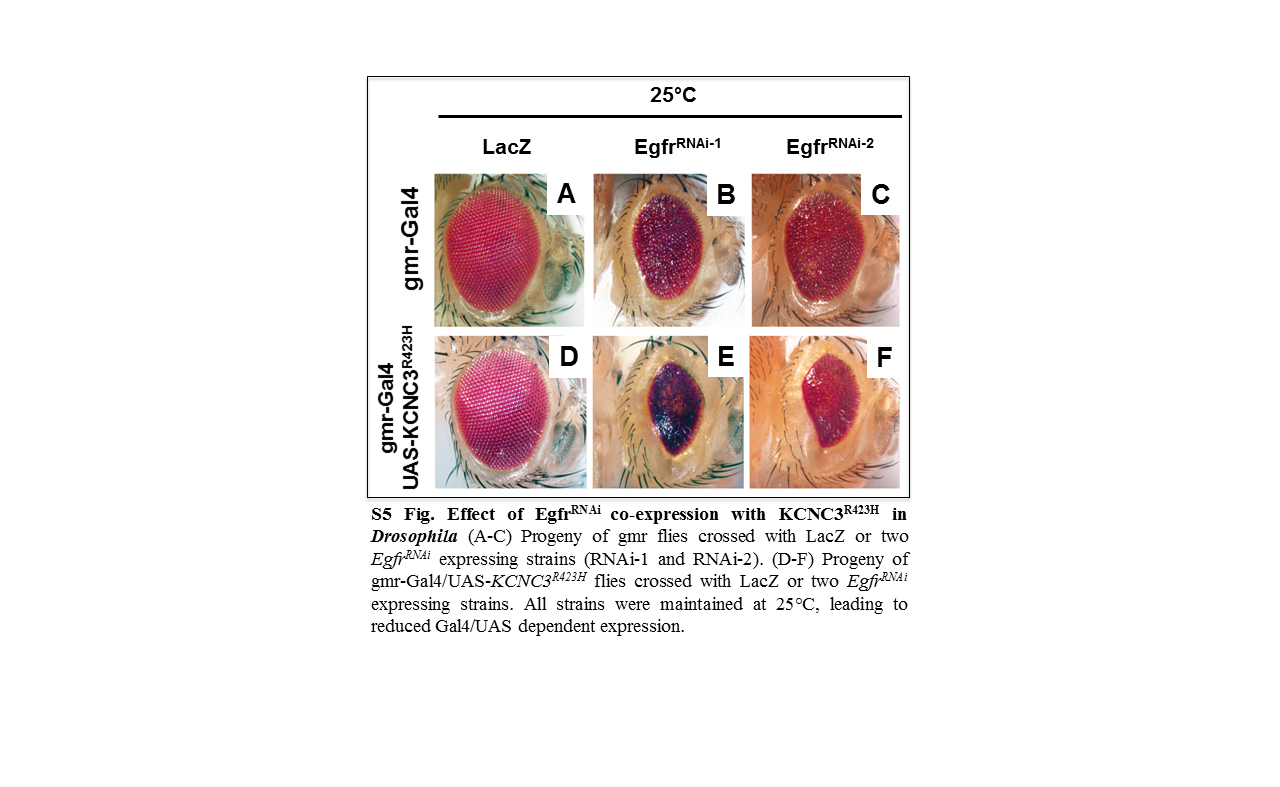

Supplement: S5 Fig — (TIF) [file pone.0173565.s010.TIF]

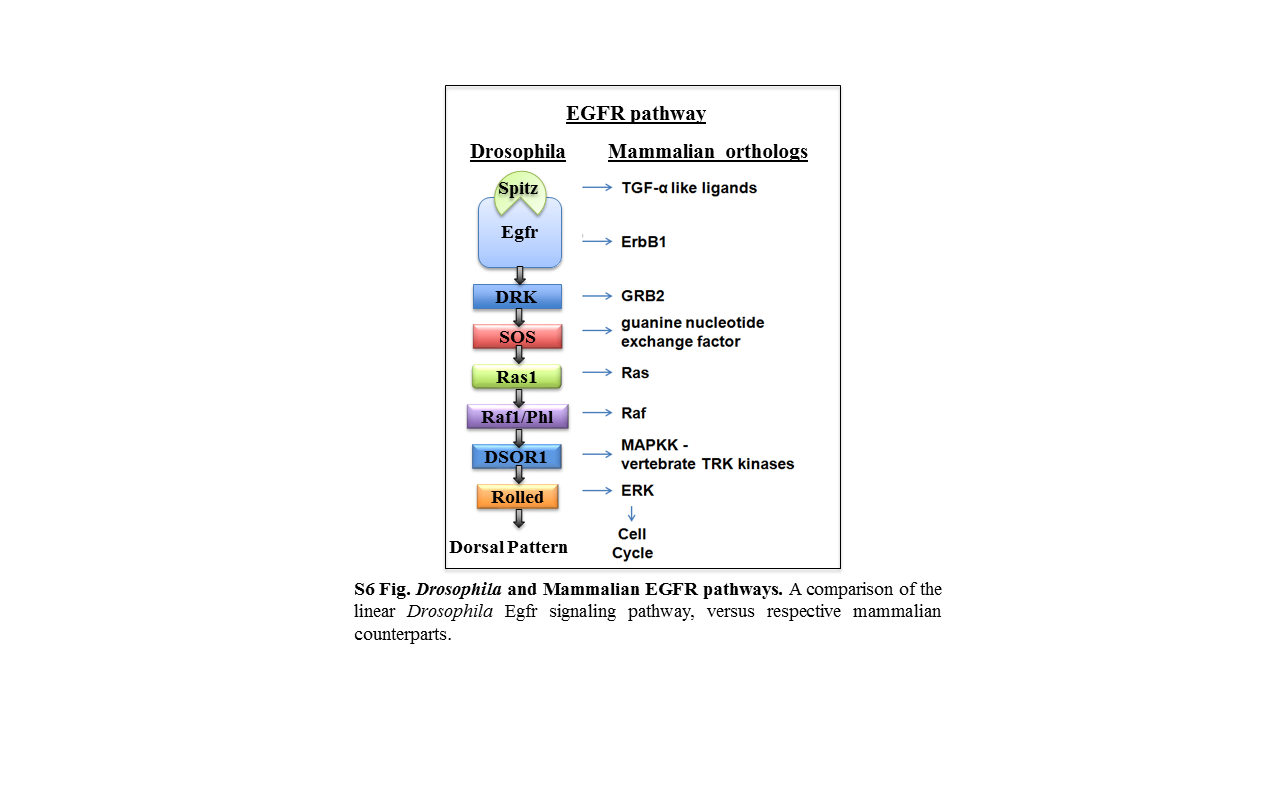

Supplement: S6 Fig — (TIF) [file pone.0173565.s011.TIF]

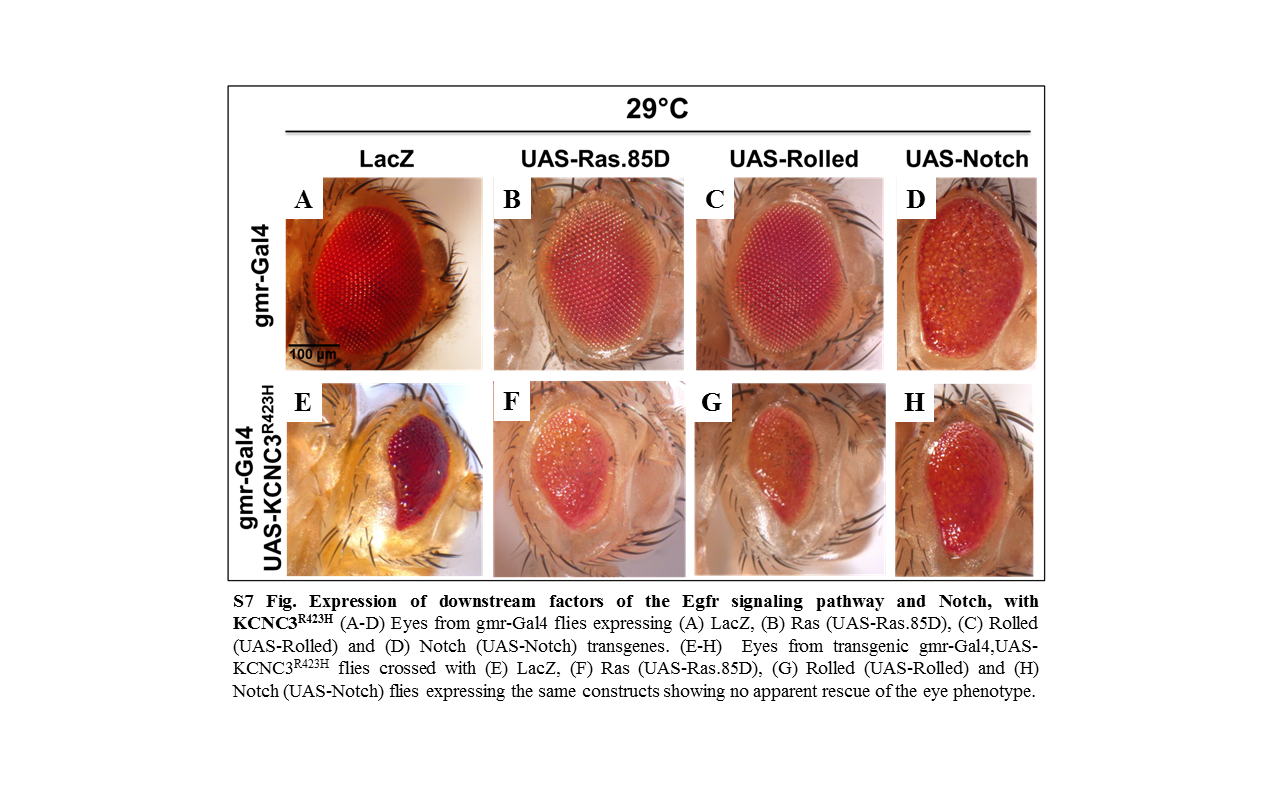

Supplement: S7 Fig — (TIF) [file pone.0173565.s012.TIF]

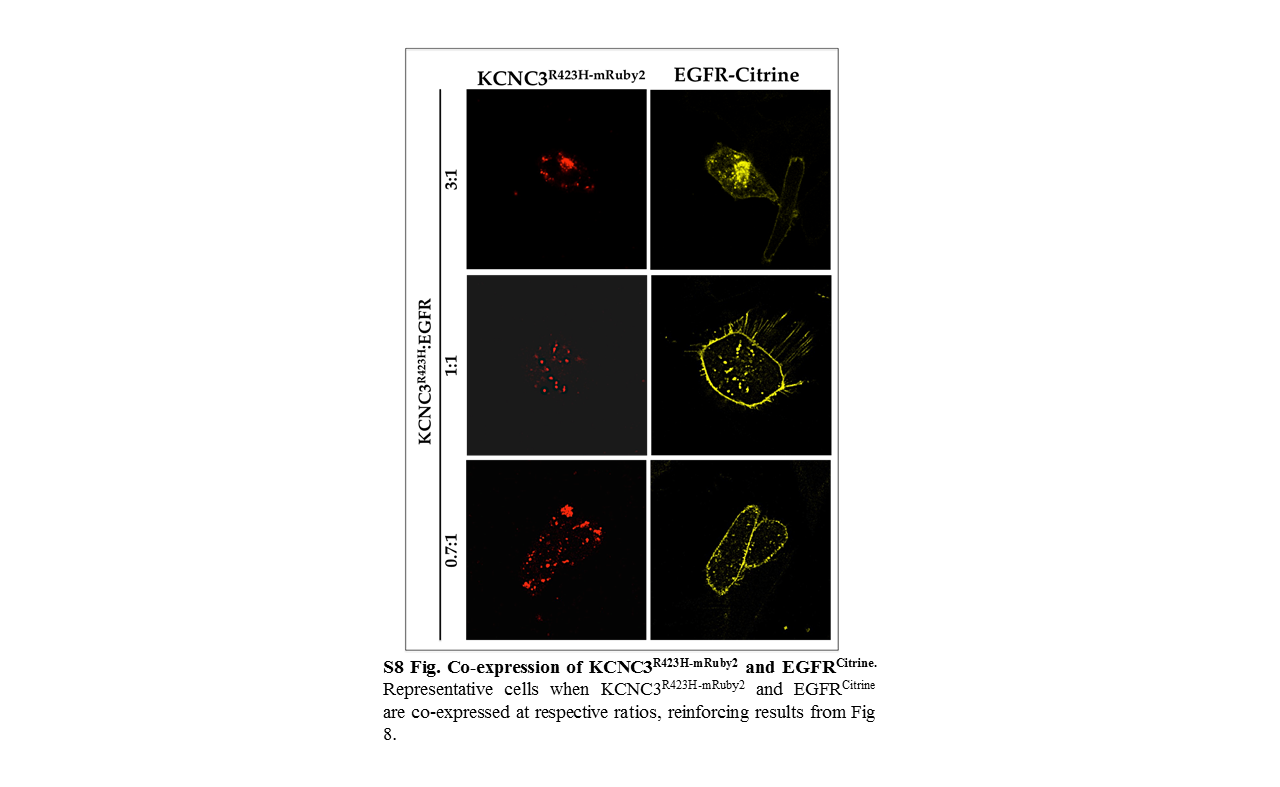

Supplement: S8 Fig — (TIF) [file pone.0173565.s013.TIF]

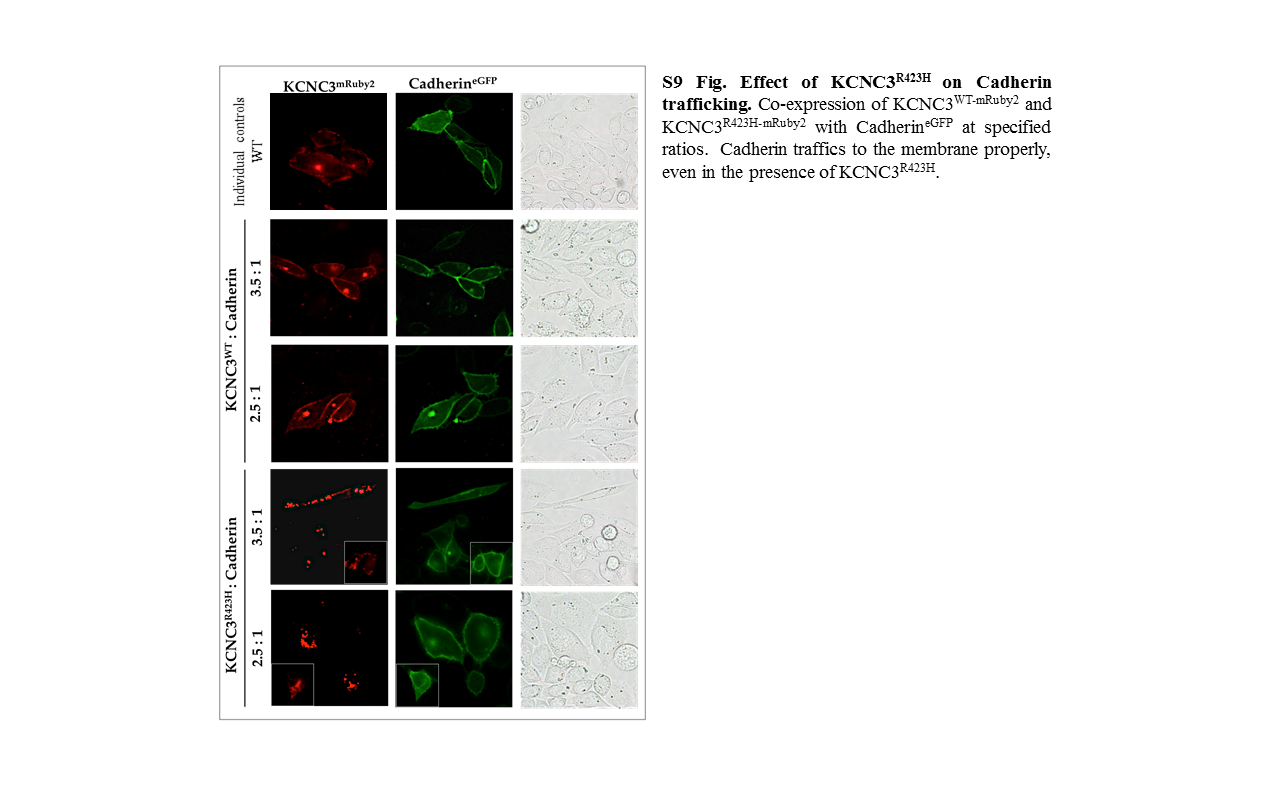

Supplement: S9 Fig — (TIF) [file pone.0173565.s014.TIF]
